# The Role of Antibiotic Resistance Genes in the Fitness Cost of Multiresistance Plasmids

**DOI:** 10.1128/mbio.03552-21

**Published:** 2022-01-18

**Authors:** Fredrika Rajer, Linus Sandegren

**Affiliations:** a Department of Medical Biochemistry and Microbiology, Uppsala University, Uppsala, Sweden; University of Pittsburgh

**Keywords:** antibiotic resistance, fitness cost, plasmid-mediated resistance

## Abstract

By providing the bacterial cell with protection against several antibiotics at once, multiresistance plasmids have an evolutionary advantage in situations where antibiotic treatments are common, such as in hospital environments. However, resistance plasmids can also impose fitness costs on the bacterium in the absence of antibiotics, something that may limit their evolutionary success. The underlying mechanisms and the possible contribution of resistance genes to such costs are still largely not understood. Here, we have specifically investigated the contribution of plasmid-borne resistance genes to the reduced fitness of the bacterial cell. The pUUH239.2 plasmid carries 13 genes linked to antibiotic resistance and reduces bacterial fitness by 2.9% per generation. This cost is fully ameliorated by the removal of the resistance cassette. While most of the plasmid-borne resistance genes individually were cost-free, even when overexpressed, two specific gene clusters were responsible for the entire cost of the plasmid: the extended-spectrum-β-lactamase gene *bla*_CTX-M-15_ and the tetracycline resistance determinants *tetAR*. The *bla*_CTX-M-15_ cost was linked to the signal peptide that exports the β-lactamase into the periplasm, and replacement with an alternative signal peptide abolished the cost. Both the tetracycline pump TetA and its repressor TetR conferred a cost on the host cell, and the reciprocal expression of these genes is likely fine-tuned to balance the respective costs. These findings highlight that the cost of clinical multiresistance plasmids can be largely due to particular resistance genes and their interaction with other cellular systems, while other resistance genes and the plasmid backbone can be cost-free.

## INTRODUCTION

Human use of antibiotics during the last 80 years has led to the selection and spread of an increasing pool of pathogenic bacteria resistant to antibiotics, with a reduction in available treatment options as a consequence. To counteract the spread of multiresistant bacteria, it is important to understand the underlying evolutionary mechanisms that drive the emergence, persistence, and dissemination of particularly successful resistant clones. One important factor is the potential fitness cost that the antibiotic resistance mechanism imposes on the bacterial cell (1). A fitness cost of resistance introduces a trade-off where in the presence of antibiotics, a resistant strain will outcompete susceptible bacteria, while in an environment without antibiotics, the resistant strain will have a disadvantage compared to an otherwise identical but susceptible strain (1–3). Most studies on fitness costs of antibiotic resistance have focused on the effects and mechanisms of chromosomal resistance mutations (4). However, less is understood about deleterious effects caused by the introduction of resistance genes carried on plasmids (5), even though such effects were identified soon after resistance plasmids were found and have been studied since (see reference 6 and references therein). Multiresistance plasmids have become an immense problem in the treatment of various infections, and they are an important factor for the dissemination of antibiotic resistance genes (7). Plasmids found in the clinic often carry a multitude of resistance genes, suggesting that the broad and frequent selection of resistance maintains these plasmids in the bacterial population (8).

It could be speculated that plasmid-borne resistance should come with a higher cost than resistance by chromosomal mutations since plasmids carry many more genes than just those involved in resistance (9). However, a meta-analysis of 77 scientific reports on the cost of resistance showed clearly that in general, *de novo* chromosomal resistance comes with a higher cost than plasmid-borne resistance, possibly since chromosomal resistance mutations often affect highly conserved genes with functions vital to the cell (4, 10). In addition, most resistance plasmids encode resistance to multiple antibiotic classes, so the cost per resistance trait becomes even lower.

Many studies have shown that alterations on both the plasmid and the bacterial chromosome can render a costly plasmid nearly cost-free and, in some cases, even beneficial (6, 8, 11–20), indicating that interactions between plasmid factors and host cell factors play a part in determining the cost and that a net cost is not an inevitable result of resistance plasmid presence even in the absence of antibiotics (6, 8, 11–23). Examples of identified genetic changes on plasmids that reduce the cost of carriage include alterations in the conjugational machinery (12, 24, 25) and the loss of plasmid-borne resistance genes (12, 25–28). In a high-throughput study of the fitness effects of resistance genes, Porse et al. found a wide variety of fitness effects when cloning 200 potential resistance genes (29). Previous studies from our laboratory have also shown that gene amplifications of resistance genes, when selecting for increased resistance, lead to a concomitant increase in the cost of the plasmid, indicating that part of the cost is due to the expression of resistance genes (30, 31). However, the exact mechanistic reasons for these fitness costs are still mostly unclear.

Here, we present a detailed analysis of the genetic determinants underlying the fitness cost of a multiresistance plasmid on its bacterial host. By using targeted deletions of individual genes or gene clusters and high-resolution competitive-fitness measurements combined with global transcriptomics and whole-cell proteomic analyses, we pinpoint the individual effects of each resistance gene on the plasmid cost and elucidate gene expression changes occurring in the bacterial cell when the multiresistance plasmid was introduced.

## RESULTS

### Large clinical multiresistance plasmids impose a small cost on bacterial fitness.

**FIG 1:**
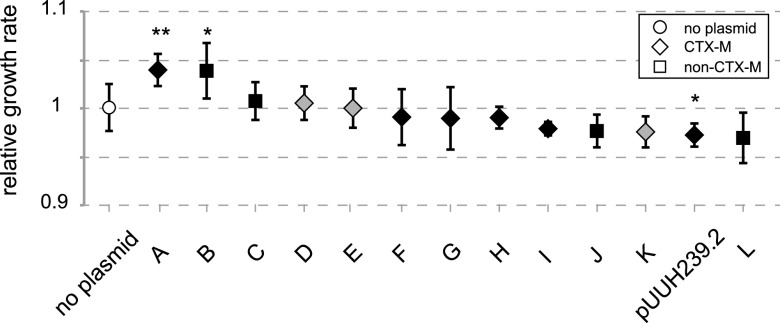
Relative growth rates of E. coli MG1655 transconjugants with different clinical plasmids. The value for the parental strain (containing no plasmid) is set to 1, and all growth rates of the plasmid-carrying strains are relative to that. Gray indicates strains carrying IncI plasmids, and black indicates IncF plasmids. Error bars denote standard deviations from 4 biological and 2 technical replicates. Statistical analysis was performed by one-way analysis of variance (ANOVA) adjusted using Dunnett’s test (*, adjusted *P* value of <0.05; **, adjusted *P* value of <0.01).

To compare the fitness costs of a set of resistance plasmids in an otherwise isogenic background, we conjugated resistance plasmids from clinical isolates of extended-spectrum-β-lactamase (ESBL)-producing Escherichia coli and Klebsiella pneumoniae to the same recipient strain (see Table S1 in the supplemental material). The resulting transconjugants were subjected to maximum-growth-rate measurements in rich medium as a proxy for the effect of the plasmids on host fitness. *In vitro* measures of growth rate or competition between strains have been found to correlate well with relative *in vivo* fitness (4), and even though differences between media may occur, it was previously shown that the relative fitness effects of a range of plasmids were maintained in different media (32). The transferred plasmids belonged to the IncF (IncFIA, IncFIB, and IncFII) or IncI group (Table 1). Three of the 13 transconjugants tested had acquired more than one plasmid from the donor through coconjugation. Most of the transferred plasmids were neutral or just slightly detrimental (up to a 3% reduction in the growth rate) to their new host (Fig. 1), but in two cases, the acquisition of the plasmid actually increased the bacterial growth rate. There was a strong correlation between the size of the plasmid and the number of resistance genes carried (Fig. S2), with some of the plasmids carrying up to 14 resistance genes. However, no correlation was observed between the size of the plasmid, or the number of resistance genes, and the relative growth rate of the bacterium, even though the latter has been found in other studies (4). The plasmid with the highest cost was pUUH239.2, originally isolated in our laboratory from a multiresistant K. pneumoniae isolate that caused an outbreak at Uppsala University Hospital in Sweden (30, 33). The numerous resistance genes carried on the plasmid as well as the fitness cost imposed by pUUH239.2 made it a suitable candidate for further in-depth analysis of the determinants underlying the associated fitness cost.

**TABLE 1:** Clinical plasmids, sizes, Inc groups, and resistance genes^*a*^

Plasmid ID	GenBank accession no.	Plasmid size (bp)	Incompatibility group(s)	Resistance gene(s)
A	CP029578	70,208	IncFII	***bla*_CTX-M-14_**
B	CP076059	214,655	IncFII, IncFIB	*aadA5*, *aac(6*′*)-Ib*, *aac(3)-IId*, *aph(6)-Id*, *aph(3*″*)-Ib*, ***bla*_TEM-1B_**, *mph*(A), *sul1*, *sul2*, *tet*(A), *dfrA17*, *cmlA1*
C	CP023821	113,737	IncFII, IncFIB	*aadA5*, *mph*(A), *sul1*, *dfrA17*
	CP023823	34,371	ND	None
D	CP076054	93,828	IncI	***bla*_CTX-M-1_**
E	CP076055	123,200	IncI	*aph(6)-Id*, *aph(3″)-Ib*, ***bla*_CTX-M-65_**, *fosA3*
F	CP023850	138,672	IncFII, IncFIA	*aadA5*, *aac(3′)-IIa*, *aac(6′)-Ib-cr*, ***bla*_CTX-M-15_**, ***bla*_OXA-1_**, *mph*(A), *sul1*, *tet*(A), *dfrA17*, *catB3*
G	CP029580	178,078	IncFII, IncFIA, IncFIB	*aadA5*, *aph(6′)-Id*, *aph(3″)-Ib*, *aac(3)-IId*, *aac(6′)-Ib*, ***bla*_TEM-1B_**, ***bla*_CTX-M-14_**, *mph*(A), *sul1*, *sul2*, *tet*(A), *dfrA17*, *ermB*, *cmlAI*
H	CP076059	214,655	IncFIB, IncFII	*aadA5*, *aac(6′)-Ib*, *aac(3)-IId*, *aph(6)-Id*, *aph(3″)-Ib*, ***bla*_TEM-1B_**, *mph*(A), *sul1*, *sul2*, *tet*(A), *dfrA17*, *cmlA1*
	CP076058	70,362	IncFII	***bla*_CTX-M-14_**
I	CP023845	181,324	IncFIA, IncFIB, IncFII	*aadA2*, *aph(3′)-Ia*, *aac(6′)-Ib-cr*, ***bla*_TEM-1B_**, ***bla*_OXA-1_**, ***bla*_CTX-M-15_**, *mph*(A), *sul1*, *tet*(A), *dfrA12*, *catB3*
	CP023846	33,067	IncX4	None
	CP023848	5,167	ND	None
J	CP076057	120,656	IncFIA, IncFII	*aadA5*, ***bla*_TEM-1B_**, *mph*(A), *sul1*, *dfrA17*
K	CP076056	89,226	IncI1	***bla*_CTX-M-14_**
pUUH239.2	NC_016966	220,824	IncFII	***bla*_TEM-1B_**, ***bla*_CTX-M-15_**, ***bla*_OXA-1_**, *mph*(A), *sul1*, *aadA2*, *aac(6′)-Ib-cr*, *dhfrXII*, *tet*(A)
L	CP029577	139,190	IncFII, IncFIB	*aadA5*, *aph(6)-Id*, *aph(3″)-Ib*, *aac(3)-IId*, ***bla*_TEM-1B_**, *mph*(A), *sul1*, *sul2*, *tet*(A), *dfrA17*

a Boldface type highlights the β-lactamase genes. ND, not determined.

10.1128/mbio.03552-21.3 FIG S2 Correlation among the size of the plasmid, the number of resistance genes, and relative fitness. In this analysis, strain H was excluded since it carried two resistance plasmids. Download FIG S2, EPS file, 1.3 MB. Copyright © 2022 Rajer and Sandegren. 2022 Rajer and Sandegren. https://creativecommons.org/licenses/by/4.0/ This content is distributed under the terms of the Creative Commons Attribution 4.0 International license.

10.1128/mbio.03552-21.5 TABLE S1 Bacterial strains and genotypes. Download Table S1, DOCX file, 0.03 MB. Copyright © 2022 Rajer and Sandegren. 2022 Rajer and Sandegren. https://creativecommons.org/licenses/by/4.0/ This content is distributed under the terms of the Creative Commons Attribution 4.0 International license.

### Resistance genes are responsible for most of the plasmid fitness cost.

**FIG 2:**
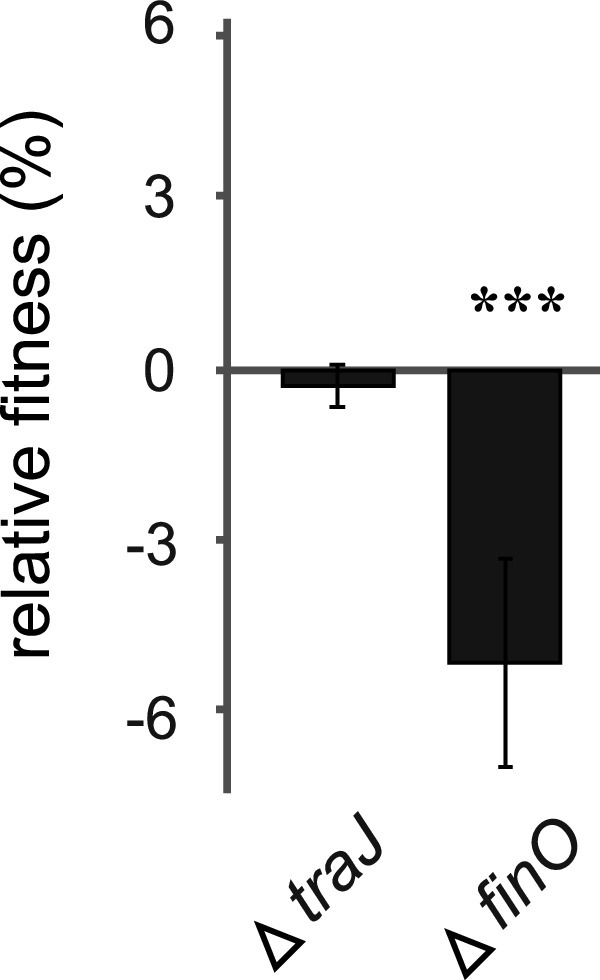
Relative fitness of mutants with altered conjugation efficiency. Error bars denote standard deviations from 16 biological replicates, including dye swaps. Statistical analysis was performed by one-way ANOVA adjusted using Dunnett’s test (***, adjusted *P* value of <0.001).

Evolution experiments have shown that a loss or reduction of the conjugational abilities can reduce the cost of plasmids (12, 24, 25). To test the contribution of conjugation to the cost of pUUH239.2, we deleted *finO*, encoding a negative regulator of conjugation (34), and the conjugation initiator *traJ* (35). This created a hyperconjugating strain (Δ*finO*) and a nonconjugating strain (Δ*traJ*). The deletion of *finO* led to a 5-log increase in the conjugation frequency and a concomitant decrease of 5% in competitive fitness (Fig. 2). The removal of the ability to conjugate by the deletion of *traJ* had no effect on fitness, which is likely explained by the already low conjugation frequency (10^−9^ per donor) of the native pUUH239.2 (30).

**FIG 3:**
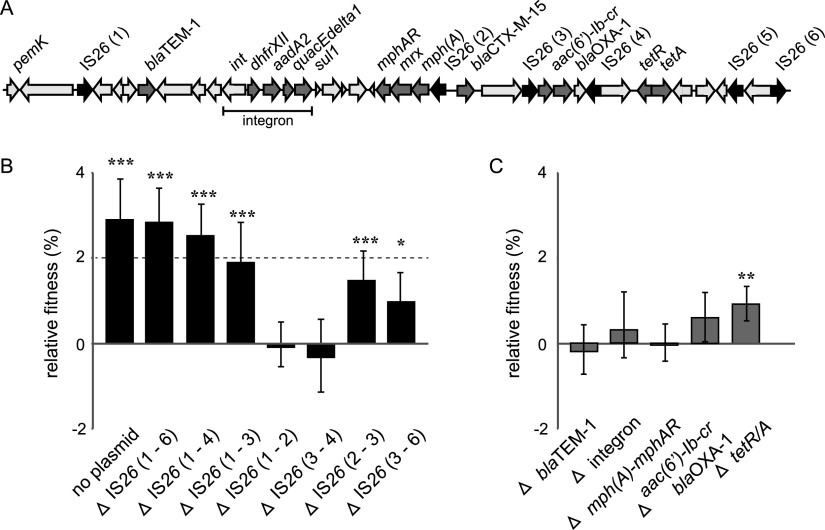
(A) Resistance region of pUUH239.2 (41 kb). Black arrows display IS*26* elements, dark gray arrows display resistance genes, and nonlabeled arrows demonstrate transposase genes (*tnp*) or other nonresistance genes. (B) Relative fitness of deletion mutants compared to the native pUUH239.2 plasmid. (C) Relative fitness of targeted deletion mutants compared to the native pUUH239.2 plasmid. Error bars denote standard deviations from at least 20 biological replicates, including dye swaps. Statistical analysis was performed by one-way ANOVA adjusted using Dunnett’s test (*, adjusted *P* value of <0.05; **, adjusted *P* value of <0.01; ***, adjusted *P* value of <0.001).

We have previously shown that increased gene copy numbers of plasmid-borne resistance genes can generate higher resistance levels but simultaneously reduce the fitness of the bacterial host (31). The resistance range (resistance to a number of different antibiotic families) is also correlated with increased cost, indicating that the actual resistance genes bear part of the cost of resistance plasmids (4). The resistance region of pUUH239.2 spans 41 kbp and contains 13 genes associated with resistance to β-lactams (*bla*_TEM-1_, *bla*_OXA-1_, and *bla*_CTX-M-15_), macrolides [*mphA*, *mrx*, and *mphR*(A)], sulfonamides (*sul1*), trimethoprim (*dhfrXII*), quaternary ammonium compounds (*quacEdelta*), aminoglycosides [*aadA2* and *aac(6′)-Ib-cr*], and tetracyclines (*tetAR*), in addition to a number of intact and partial transposase genes (Fig. 3A). The resistance region is flanked by two identical IS*26* elements, and four additional IS*26* elements are interspersed among the resistance genes. To investigate the contribution of resistance genes to the fitness cost of pUUH239.2, we constructed a complete deletion of the resistance region [ΔIS*26*(1–6)] as well as randomized deletions of shorter regions among the six IS*26* elements, resulting in a collection of different deletions spanning all resistance genes individually or in combination. The deletion of the entire resistance region [IS*26*(1–6)] abolished the plasmid cost completely (Fig. 3B). Deletions of regions containing *bla*_TEM-1_, *sul1*, *aadA2*, *dhfrXII*, *mphRA-mrx*, and *quacEdelta* [IS*26*(1–2)] or *bla*_OXA-1_ and *aac(6′)-Ib-cr* [IS*26*(3–4)] showed no change in fitness, but deletion of the region containing the ESBL gene *bla*_CTX-M-15_ [IS*26*(1–3)] increased fitness by 1.9%, and deletion of the region containing *bla*_OXA-1_, *aac(6′)-Ib-cr*, and *tetRA* [IS*26*(3–6)] increased fitness by 1.2%. Precise deletion mutants were also constructed where resistance genes were removed individually or in smaller clusters but all other genes were left intact. As with the larger deletions, the individual removal of *bla*_TEM-1_, *sul1*, *aadA2*, *dhfrXII*, *mphRA-mrx*, *aac(6′)-Ib-cr*, and *bla*_OXA-1_ did not result in a significant reduction of the fitness cost, while deletion of the *tetAR* genes again increased fitness by 1% (Fig. 3C). Unfortunately, a targeted deletion of *bla*_CTX-M-15_ alone could not be constructed even after numerous attempts. However, deletion of the region between IS*26*(2) and IS*26*(3), including *bla*_CTX-M-15_ and one transposase gene, increased fitness by 1.5% compared to the native plasmid (Fig. 3B), again confirming the involvement of this region in the fitness cost of the plasmid.

### *bla*_CTX-M-15_ is one of the most highly expressed genes in the cell during exponential growth.

**FIG 4:**
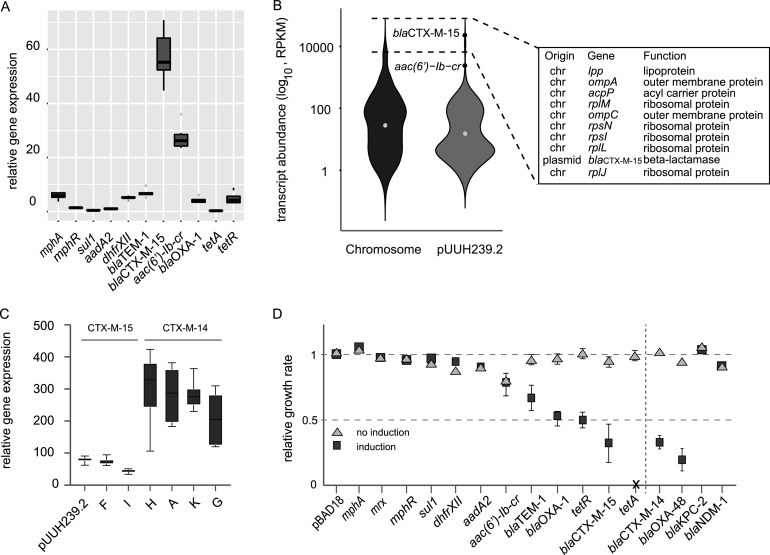
(A) RT-qPCR data of antibiotic resistance genes from the pUUH239.2-containing strain. The gene expression of the resistance genes is relative to those of the housekeeping genes *cysG* and *hcaT*. Error bars denote the standard deviations from three biological and three technical replicates. (B) Violin plot representing the distribution of transcript abundances (in log_10_ reads per kilobase per million [RPKM]) of chromosomal (dark gray) and plasmid pUUH239.2 (light gray) genes. The dots denote the median values of the distribution. The resistance gene *bla*_CTX-M-15_ is one of the top 10 most-expressed genes. chr, chromosomal. (C) Relative gene expression of the β-lactamase genes *bla*_CTX-M-15_ and *bla*_CTX-M-14_ from clinical plasmids. Error bars denote the standard deviations from three biological and three technical replicates. (D) Effect on the maximum growth rate by the overexpression (by the addition of 0.05% l-arabinose) of resistance genes from pBAD18 expression vectors. All values are relative to the value for the empty vector, which is set to 1. Error bars denote the standard deviations from 4 biological replicates.

Since a gene most likely is costly only if it is expressed and the expression level correlates with the fitness cost, we determined the expression levels of all resistance genes from their native promoters by quantitative PCR and the complete transcription profile of the cell by transcriptome sequencing (RNASeq) (Fig. 4; Table S3). The relative expression levels of resistance genes differed greatly, with *bla*_CTX-M-15_ being the most highly expressed during exponential growth. However, *tetA* had the lowest expression level, in accordance with it being suppressed by TetR, indicating that this low residual expression of *tetA* or the basal expression of *tetR* gives a significant cost to the cell (Fig. 4A). Interestingly, *bla*_CTX-M-15_ was among the top 10 most-expressed genes in the cell, along with ribosomal genes and outer membrane proteins (Fig. 4B). To verify if the high expression of *bla*_CTX-M-15_ is a general feature or specific for pUUH239.2, we measured the steady-state expression of *bla*_CTX-M-15_ and *bla*_CTX-M-14_ from the transconjugants containing clinical plasmids. All tested *bla*_CTX-M-15_ genes were expressed to the same level as from pUUH239.2, while the *bla*_CTX-M-14_ genes were even more highly expressed (Fig. 4C).

10.1128/mbio.03552-21.7 TABLE S3 RPKM data for the two pUUH239.2-containing strains. The 50 most-expressed transcripts are shown. Download Table S3, DOCX file, 0.02 MB. Copyright © 2022 Rajer and Sandegren. 2022 Rajer and Sandegren. https://creativecommons.org/licenses/by/4.0/ This content is distributed under the terms of the Creative Commons Attribution 4.0 International license.

### β-Lactamases and *tetA* are the costliest resistance genes when overexpressed.

To measure the relative fitness effects of the individual resistance genes with equal gene expression levels, we cloned them behind an l-arabinose-inducible promoter on the pBAD18 vector (36). Induction by l-arabinose had the most severe effect on growth for strains expressing *bla*_CTX-M-15_, *tetA*, and *tetR*, in agreement with the deletion data, but *bla*_TEM-1_ and *bla*_OXA-1_ also reduced fitness when overexpressed (Fig. 4D). However, *mphA*, *mrx*, *mphR*(A), *sul1*, *dhfrXII*, and *aadA2* did not give any reduction in fitness, indicating that these plasmid-borne resistance genes do not affect their host in a detrimental way even when overexpressed from a multicopy plasmid. Validation of the functionality of the cloned genes was done by MIC tests (Table S4).

10.1128/mbio.03552-21.8 TABLE S4 Susceptibility testing (milligrams per liter) of pBAD18 expression vectors containing cloned resistance genes. Tryptone broth agar with the addition of 50 μg/mL kanamycin and 0.05% l-arabinose was used. Values denote the medians from three biological replicates. Download Table S4, DOCX file, 0.02 MB. Copyright © 2022 Rajer and Sandegren. 2022 Rajer and Sandegren. https://creativecommons.org/licenses/by/4.0/ This content is distributed under the terms of the Creative Commons Attribution 4.0 International license.

Even though the three β-lactamases tested are of different genetic families, they all clearly had a detrimental effect when overexpressed. To test the generality of β-lactamase costs, we included an additional set of clinically prevalent β-lactamases with catalytic activity against different β-lactam groups (Fig. 4D; Table S4). The ESBL CTX-M-14 as well as the carbapenemase OXA-48 conferred the same drastic decrease in growth as CTX-M-15 when overexpressed, whereas the carbapenemases NDM-1 and KPC-2 were neutral to the host. This is in contrast to previous studies where cloned *bla*_OXA-48_ was not associated with a fitness cost (37, 38).

### The CTX-M-15 signal peptide is highly costly.

**FIG 5:**
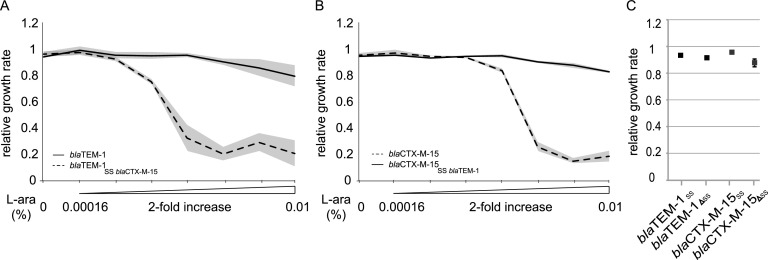
(A and B) Relative growth rates of *bla*_TEM-1_ (A), *bla*_CTX-M-15_ (B), as well as hybrid genes with an exchanged signal sequence (SS) when overexpressed from pBAD18. (C) Relative growth rates of cells expressing only the signal sequence of the β-lactamases or the β-lactamases without the signal sequence. Induction was performed with 0.01% l-arabinose, and values for all strains are relative to those for the empty pBAD18 expression vector. Error bars denote standard deviations from 4 biological replicates.

A previous study showed that the signal peptide of the β-lactamase SME-1 had a negative impact on the growth rate of the cell (26). The β-lactamase signal peptide ensures the proper translocation of the enzyme to the periplasmic space, where the enzyme then protects the cell by degrading the antibiotic (39, 40). To investigate if the signal peptide of CTX-M-15 was involved in the fitness cost, we constructed gene variants where the signal peptides were interchanged between *bla*_TEM-1_ (low cost) and *bla*_CTX-M-15_ (high cost). The induction of the native *bla*_TEM-1_ led to a small reduction in the growth rate at the highest concentration of l-arabinose used (Fig. 5A), whereas the native *bla*_CTX-M-15_ (Fig. 5B) reduced the growth rate drastically already at much lower levels of induction. Exchanging the signal peptides completely reversed the growth defects. Expressing only the signal peptides or the β-lactamases without signal peptides did not affect bacterial growth (Fig. 5C). Exchanging the signal peptides did not change the MIC for the two β-lactamases, indicating that enzymatic transfer to the periplasm still occurred (Table S4). The MIC has been shown previously to be a very good reflection of the total amount of β-lactamase activity in the periplasm (41). The removal of the signal peptide abolished TEM-1 activity but still enabled decreased susceptibility to cefotaxime by the CTX-M-15 enzyme, indicating low-level transfer of the CTX-M-15 enzyme to the periplasm even without a signal peptide, in agreement with previous findings for other β-lactamases (42, 43).

### TetA and TetR expression is balanced to reduce the fitness cost.

Since the combined removal or individual overexpression of the TetA efflux pump and the repressor TetR affected fitness, we further evaluated their individual cost contribution in the native genetic context. In order to not introduce downstream effects on the overlapping promoters and regulatory binding sites, a flippase (FLP) recombination target (FRT) scar (causing a frameshift) was introduced into each reading frame, destroying the activity of the respective proteins. As expected, disruption of *tetR* resulted in a tetracycline MIC identical to that of the native pUUH239.2, whereas disruption of *tetA* reduced the MIC to the plasmid-free level (Table S4). Disruption of *tetA* also led to an increase in fitness of 0.5%, indicating that the gene is not completely repressed even in the absence of tetracycline under the tested growth conditions (Fig. S3A). In contrast, disruption of *tetR* led to an additional fitness cost of 1.2% compared to the native pUUH239.2 and an expression level of *tetA* similar to that with induction with autoclaved chlortetracycline (which does not have antibiotic activity but induces the expression of *tetA* [44]) (Fig. S3B). This suggests that the cost observed in the competition assays is due to a balance between having enough TetR expression to repress *tetA* in the absence of tetracycline and at the same time avoiding the high cost of TetR expression.

10.1128/mbio.03552-21.4 FIG S3 (A) Relative fitness of pUUH239.2 with inactivated *tetA* and *tetR* genes. Error bars denote standard deviations from eight biological replicates, including dye swap. Statistical analysis was performed by one-way ANOVA adjusted using Dunnett’s test (***, adjusted *P* value of <0.001). (B) Relative expression of *tetA*, *tetR*, and inactivated versions from pUUH239.2 in the absence and presence of autoclaved chlortetracycline (aCT). Error bars represent data from 3 biological and 2 technical replicates. Download FIG S3, EPS file, 1.3 MB. Copyright © 2022 Rajer and Sandegren. 2022 Rajer and Sandegren. https://creativecommons.org/licenses/by/4.0/ This content is distributed under the terms of the Creative Commons Attribution 4.0 International license.

### Differential gene expression and protein abundance due to the pUUH239.2 plasmid.

Although we have pinpointed here the fitness cost of the pUUH239.2 resistance plasmid to the balance of the expression of the *tet* genes and the signal peptide of *bla*_CTX-M-15_, the actual mechanistic cause of the cost remains unexplained. To address this, we looked at global changes in gene expression and protein abundance upon the introduction of the original pUUH239.2 and versions with deletions of the whole resistance cassette, *bla*_CTX-M-15_ or *tetRA*, respectively. Surprisingly, only minor changes in chromosomal gene expression were observed between the pUUH239.2-containing strain and a plasmid-free strain (Table S5). The chromosomal gene with the largest increase in expression was *insO* (putative prophage-related gene), with a 5-fold increase, and the one with the largest reduction was *glnK* (regulation during nitrogen starvation) (45), with a 16-fold decrease. Most transcriptional changes were below 2-fold and largely followed by the respective protein levels (Table 2). Functional clustering of the genes affected by changed expression showed a pattern of a slight upregulation of genes involved in microaerobic respiration, like *cydA* and *cydB* (46), and a slight downregulation of genes involved in oxidative respiration, such as *cyoABCD* (Table S6). However, on the protein level, there were four proteins that displayed striking increases in abundance in the presence of the plasmid: a 29-fold increase of YbdM (hypothetical protein), 13- and 18-fold-increased levels of TorA/TorC (anaerobic respiration), and a 5-fold increase of MmuP (*S*-methylmethionine transporter) (Table 2). Interestingly, these changes were all connected to the presence of the resistance cassette, and the changes in YbdM and MmuP were directly connected to the presence of *bla*_CTX-M-15_. No specific differences in protein abundance were connected to *tetAR*.

**TABLE 2:**
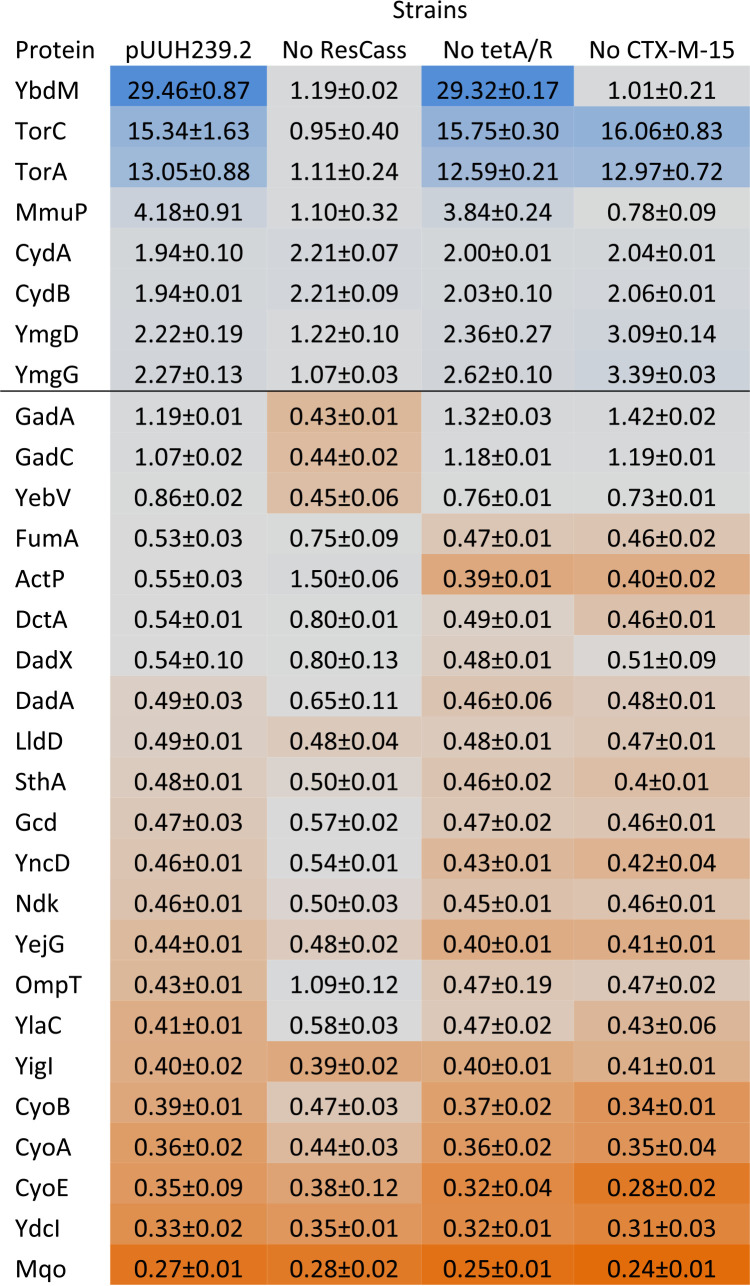
Changes in protein abundance when carrying pUUH239.2^*a*^

a All values are relative to the parental strain. Means ± standard deviations from 2 biological replicates are shown. ResCass, strain carrying pUUH239.2 without the resistance cassette. Colors indicate fold change of the chromosomally expressed proteins. Blue is more abundant and orange is less abundant protein.

10.1128/mbio.03552-21.9 TABLE S5 Transcriptomics data (in duplicates) of plasmid-containing strains and significant up- and downregulated chromosomal genes compared to plasmid-free cells. Values are on a log_2_ scale. Download Table S5, DOCX file, 0.04 MB. Copyright © 2022 Rajer and Sandegren. 2022 Rajer and Sandegren. https://creativecommons.org/licenses/by/4.0/ This content is distributed under the terms of the Creative Commons Attribution 4.0 International license.

10.1128/mbio.03552-21.10 TABLE S6 Enriched KEGG pathways of a strain carrying pUUH239.2 compared to a strain without the plasmid. Download Table S6, DOCX file, 0.02 MB. Copyright © 2022 Rajer and Sandegren. 2022 Rajer and Sandegren. https://creativecommons.org/licenses/by/4.0/ This content is distributed under the terms of the Creative Commons Attribution 4.0 International license.

**FIG 6:**
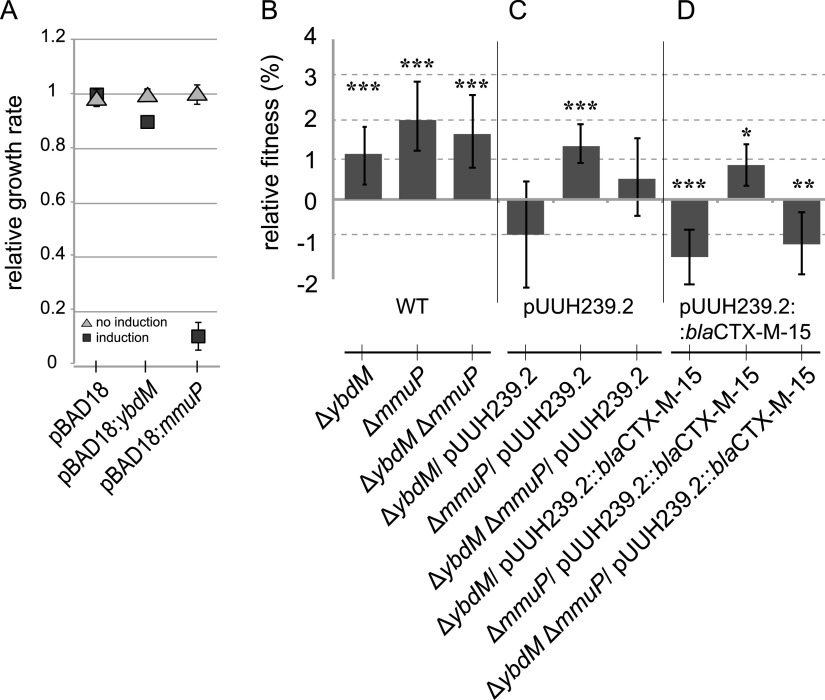
(A) Relative growth rates of strains with pBAD18 vectors containing *ybdM* or *mmuP* induced with 0.01% l-arabinose. Error bars denote standard deviations from four biological replicates. (B) Relative fitness of E. coli MG1655 mutants with deletions of *ybdM* and *mmuP* compared to the parental strain. WT, wild type. (C) Relative fitness of E. coli MG1655 mutants with deletions of *ybdM* and *mmuP* carrying pUUH239.2 compared to the parental strain carrying native pUUH239.2. (D) Relative fitness of E. coli MG1655 mutants with deletions of *ybdM* and *mmuP* carrying pUUH239.2::*bla*CTX-M-15 compared to the parental strain carrying pUUH239.2::*bla*CTX-M-15. Competition assays were performed with at least 8 biological replicates, including dye swaps. Statistical analysis was performed by one-way ANOVA adjusted using Dunnett’s test (*, adjusted *P* value of <0.05; **, adjusted *P* value of <0.01; ***, adjusted *P* value of <0.001).

To investigate the possible connection of YbdM and MmuP to the cost of CTX-M-15, we tested the effects of deletion and overexpression of the two genes. The overexpression of YbdM gave a 10% reduction in the growth rate, while overexpression of MmuP gave an almost complete growth arrest of the cells (Fig. 6A). Deletion of either *ybdM*, *mmuP*, or the combination of both led to an increase in fitness of 1 to 2% in the absence of the plasmid (Fig. 6B). Interestingly, combined with pUUH239.2, Δ*ybdM* instead gave a net reduction in fitness of 1%, while Δ*mmuP* gave an advantage similar to that without the plasmid (Fig. 6C). The double deletion combined with pUUH239.2 was neutral compared with the wild type containing pUUH239.2. However, this effect was not connected to the presence of *bla*_CTX-M-15_ (Fig. 6D). We conclude that although overexpression of YbdM and, especially, MmuP is costly to the cell, deletion of the respective genes in the presence of *bla*_CTX-M-15_ led to different outcomes, with an increase in the plasmid cost for Δ*ybdM*, while deletion of *mmuP* did not affect the cost of the plasmid. This could indicate that YbdM has a positive effect on fitness in the presence of the pUUH239.2 plasmid and that the increase in protein abundance is linked to this. However, these fitness differences were maintained even in the absence of *bla*_CTX-M-15_ and therefore do not match the protein abundance data where the increase was directly coupled to the presence of *bla*_CTX-M-15_.

## DISCUSSION

The molecular basis of fitness effects of individual plasmid-borne resistance genes on the bacterial cell in the absence of antibiotics is not well known despite this type of resistance being a major problem in clinical settings. The presence of one or multiple plasmids in clinical isolates is very common (47–49), and an inferred fitness cost on the bacterial cell would negatively affect the maintenance of the plasmid in the bacterial population (50). Our results corroborate the general picture in the literature that the carriage of natural low-copy-number resistance plasmids is largely neutral or just slightly costly to the host in most cases (4). No correlation between the growth rate and the plasmid size or number of resistance genes could be found here, but larger plasmids tended to have more resistance genes. In Vogwill and MacLean’s metastudy of resistance cost, there was a clear correlation between the cost and the number of different antibiotics that the plasmids encoded resistance to, indicating that resistance genes are more costly than the majority of plasmid-borne genes (4). Here, pUUH239.2 was one of the costliest plasmids that we tested, although the reduction in competitive growth of 2.9% is small compared to some plasmid fitness costs reported (8, 12, 20, 24).

Previous studies have found reduced conjugation to be beneficial to limit the cost displayed by plasmids (12, 24, 51). We confirmed that the conjugation of pUUH239.2 was indeed costly but that, at least under the experimental conditions tested here, conjugation seemed to be repressed to such an extent that it did not contribute to the overall cost of the plasmid. This confirms the trade-off between increased horizontal spread and stable vertical transmission and that the frequency of conjugation is of importance in maintaining a balance between the transfer frequency and the cost of conjugative plasmids (5, 24, 52–55).

The cost of pUUH239.2 was completely alleviated by the deletion of the resistance cassette, and out of the 13 resistance-associated genes, the reduction in fitness could be pinpointed to *tetAR* and *bla*_CTX-M-15_. The cost of the expression of the TetA pump is likely the reason why this gene has tightly controlled expression through the TetR repressor to be expressed only when needed. The cost of *tet* genes was detected very early (56). Nguyen et al. performed a thorough evaluation of the cost of the expression of the Tn*10* Tet region and found a strong negative effect of the expression of TetA but no effect at all of the overexpression of TetR (28). In contrast to that study, we find that even the expression of *tetR* gives rise to a reduction in fitness, resulting in a cost balance between the repression of *tetA* and the expression of the regulator that together constitute a 1% cost of the system. The *tetAR* genes on pUUH239.2 are of the *tet*(A) family, while the *tet* genes in Tn*10* belong to the *tet*(B) family, and they share <50% identity on the protein level, which could explain this discrepancy. It is unlikely that the costs of the pump and the repressor have the same molecular basis, and we do not find any clues as to the cause of why the *tetAR* system is costly in our RNA or protein data. Since we tested the system only under the native repressed conditions, the individual effects resulting in the combined 1% fitness reduction might be too small to be detected. An approach with gradual induction of the genes separately may reveal the underlying causes better.

The costliest gene was the extended-spectrum β-lactamase CTX-M-15. Whole-cell transcriptomics revealed that *bla*_CTX-M-15_ is among the top 10 most-expressed genes in the plasmid-containing cell along with ribosomal proteins. The high expression level of *bla*_CTX-M-15_ was not unique to pUUH239.2 but matched that of all clinical plasmids tested here and also *bla*_CTX-M-14_. The other two β-lactamase genes on pUUH239.2, *bla*_TEM-1_ and *bla*_OXA-1_, had much lower basal expression levels, and the deletion of these genes did not affect host cell fitness. However, the overexpression of all three β-lactamases from pUUH239.2 or the carbapenemase OXA-48 gave considerably higher costs than the overexpression of any other resistance genes from pUUH239.2 besides *tetAR*. To our knowledge, the only report of a fitness cost attributed to a β-lactamase is for the chromosomal carbapenemase SME-1 in Serratia marcescens (26). Like for SME-1, we linked the cost of CTX-M-15 to the signal peptide, and exchanging the signal peptides between CTX-M-15 and TEM-1 completely reversed the costs while still protecting the cell against antibiotics. This indicates that it is the transport of the β-lactamase to the periplasm by the CTX-M signal peptide *per se* that is invoking the cost and not the catalytic activity of the enzyme. It is likely that the effect of the signal peptide on bacterial fitness is through interference with, or occupation of, the secretory machinery that reduces the normal transfer of other proteins to the periplasm. Examination of other β-lactamases from different Ambler groups indicated a wide range of fitness effects on the host cell, with *bla*_KPC-2_ not giving any cost at all when overexpressed, while *bla*_CTX-M_ genes and *bla*_OXA-48_ gave high costs. *bla*_CTX-M_ is the most widespread family of ESBL genes (57, 58), and it is striking that this group of enzymes seems to both rely on a very high transcription level and invoke a cost of enzyme transfer to the periplasm that is not necessary for other groups of β-lactamases.

The transcriptomic data showed that the presence of the pUUH239.2 plasmid did not come with large changes in chromosomal gene expression, and the observed changes were not coupled with the presence of the resistance cassette and therefore were not further studied here. This is in contrast to a recent report where plasmid acquisition in Pseudomonas aeruginosa led to a fitness cost through large effects on chromosomal gene expression and where fitness-compensating mutations on the chromosome restored expression and fitness (20).

However, our proteomics data revealed substantial changes in protein abundance for four proteins that were directly linked to the presence of the resistance cassette. Most interestingly, the increase in YbdM and MmuP abundances was directly coupled to the presence of CTX-M-15. Unfortunately, very little is known about these two proteins. YbdM is completely uncharacterized, while MmuP has been found encoded on clinically isolated resistance plasmids and was indicated to be involved in selenium resistance (59–61), osmotic tolerance (62), and *S*-methylmethionine utilization (63). The deletion or overexpression of YbdM and MmuP in the presence and absence of pUUH239.2 did not reveal any clear clues. The elevated cost of the plasmid in the absence of YbdM could indicate that it is somehow mitigating part of the plasmid cost. However, this effect was not coupled to the presence or absence of CTX-M-15, making it unlikely that the increased protein abundance of YbdM is a way for the cell to compensate for the presence of the β-lactamase.

In conclusion, we find that the fitness cost conferred by pUUH239.2 can be fully explained by the balanced expression of *tetAR* and through the signal peptide of CTX-M-15. Our findings also show that many important resistance genes and the plasmid backbone of large plasmids can be cost-free to the bacterial cell and, hence, are likely to be stably maintained in the bacterial population even in the absence of antibiotic selection. This is unfortunate since mathematical calculations of the persistence of low-cost resistance genes after a ban of a certain antibiotic have shown that the reduction of such resistant strains will be very slow (50).

## MATERIALS AND METHODS

### Bacterial strains and growth conditions.

Clinical isolates from which resistance plasmids were transferred were reported in previous studies (47, 64). The constructed strains were derived from Escherichia coli K-12 MG1655, as listed in Table S1 in the supplemental material. Bacteria were grown in Mueller-Hinton broth (MHB; Becton, Dickinson and Company) and on Mueller-Hinton agar (MHA; Becton, Dickinson and Company) with the addition of substances or antibiotics, as stated below. The following antibiotics (Sigma-Aldrich) were used: chloramphenicol (15 mg/L), cefotaxime (10 mg/L), trimethoprim (10 mg/L), kanamycin (50 mg/L), tetracycline (15 mg/L), and zeocin (20 mg/L). For Δ*dapA* mutants, 20 mg/L 2,6-diaminopimelic acid (DAP; Sigma-Aldrich) was added to agar and liquid medium when necessary for growth.

### Strain construction.

The pUUH239.2 plasmid used in this study is a derivative of the original pUUH239.2 plasmid (GenBank accession number CP002474.1) without the original triplicated region in the resistance cassette. Genetic modifications were done in a strain carrying the λ red recombineering system on the chromosome, and deletions were done by homologous linear recombination by inserting a *cat* cassette (Table S2), as described previously (65). The *cat* cassette was subsequently removed by the transient transformation of pCP20, carrying FLP (flippase), leaving an FRT scar (65), and deletions were verified by PCR. For the complete deletion of the resistance region, the *cat* cassette was kept in order to maintain selection for the plasmid in the recipient strain. The cost of the *cat* cassette was 0.9% (Fig. S1) and was compensated for in subsequent calculations. Resistance genes were cloned into a pBAD18-kan vector by the insertion of the respective open reading frames downstream of the arabinose-inducible promoter (Table S2) (36). Alternative versions of the β-lactamases *bla*_TEM-1_ and *bla*_CTX-M-15_ with changed signal peptides were created by gene synthesis at IDT (Integrated DNA Technologies) (Table S2).

10.1128/mbio.03552-21.2 FIG S1 Cost of the *cat* cassette. Error bars denote standard deviations (SD) from 8 biological replicates, including dye swap. A *t* test was performed for statistical significance (***, *P* > 0.001). Download FIG S1, EPS file, 1.2 MB. Copyright © 2022 Rajer and Sandegren. 2022 Rajer and Sandegren. https://creativecommons.org/licenses/by/4.0/ This content is distributed under the terms of the Creative Commons Attribution 4.0 International license.

10.1128/mbio.03552-21.6 TABLE S2 Primer sequences for deletions on the pUUH239.2 plasmid and chromosome as well as for the cloning of resistance genes. Download Table S2, DOCX file, 0.02 MB. Copyright © 2022 Rajer and Sandegren. 2022 Rajer and Sandegren. https://creativecommons.org/licenses/by/4.0/ This content is distributed under the terms of the Creative Commons Attribution 4.0 International license.

### Conjugation experiments.

All conjugations were made through a Δ*dapA* strain for efficient counterselection without the need for antibiotic resistance markers in the final recipient. Cultures of the donor and Δ*dapA* recipient strains grown overnight were mixed at a 1:2 ratio, spun down, placed onto sterile filters on MHA containing DAP, and incubated overnight at 37°C. The following day, the bacteria were washed from the filter with 1× phosphate-buffered saline (PBS) (8 g/L NaCl, 0.2 g/L KCl, 1.44 g/L Na_2_HPO_4_, and 0.24 g/L KH_2_PO_4_) and plated onto MHA containing DAP, chloramphenicol, and a selective antibiotic for the plasmid to be transferred. The transconjugants obtained were used as donors for a second conjugation of the respective plasmids into the desired recipient. Transconjugants were whole-genome sequenced with an Illumina MiSeq platform to identify transferred plasmids and make sure that no additional genetic changes had occurred in the recipients.

### Whole-genome sequencing.

Transconjugants were sequenced by a combination of Oxford Nanopore and Illumina MiSeq technologies. Complete genomic DNA (gDNA) was purified using the Qiagen Genomic-Tip 100 kit according to the manufacturer’s directions. Library preparations for Nanopore sequencing were performed with the Rapid barcoding kit (Nanopore) according to the manufacturer’s recommendations. Multiplexed libraries were concentrated using AMPure XP beads (Nanopore) and sequenced in a flow cell until no active pores remained. MiSeq libraries were prepared and sequenced by 300-bp paired-end read lengths using a Nextera XT DNA library preparation kit (Illumina) according to the manufacturer’s instructions. *De novo* assembly of the Nanopore data was performed using the long-read support function in CLC Genomics Workbench v.20 (Qiagen). Contigs were reanalyzed by reference assembly using MiSeq data with CLC Genomics Workbench v.20 including the Microbial Analysis Pro suite (Qiagen). Detection of resistance genes and plasmid replicons was done by submitting *de novo*-assembled contigs to the online resources ResFinder (66) and PlasmidFinder (67), respectively, at the Center for Genomic Epidemiology, DTU, Denmark.

### Fitness cost of plasmid carriage.

Maximum growth rates were measured at 37°C by using a BioscreenC MBR (Oy Growth Curves Ad Ltd.), taking optical density at 600 nm (OD_600_) measurements every 4 min. The maximum growth rate was based on the OD_600_ values where growth was observed to be exponential. For E. coli MG1655 with clinical plasmids, MHB was used. Since arabinose induction is inhibited by glucose (36), growth rates of the strains carrying pBAD18 expression vectors were measured in tryptone broth at 37°C. The induction of genes from pBAD18 was done by the addition of 0.01% or 0.05% l-arabinose, and 50 mg/L kanamycin was included to select for the plasmid.

For competition experiments, bacteria expressing SYFP2 (68) or mTagBFP2 (69) were used. Cultures of the competing strains grown overnight were mixed at a ratio of 1:1 in fresh MHB and passaged every 24 h by 1,000-fold dilution into fresh medium, resulting in approximately 10 generations of growth per passage. Each competition was run for 30 generations. By counting 10^5^ competing cells at each serial passage using a MACSQuant system (Miltenyi Biotec), the ratio between competing strains was obtained. The regression model *s* = {ln[*R*(*t*)/*R*(0)]}/[*t*] was used to calculate the selection coefficient in each experiment, where *R* is the ratio between the strain with no plasmid or an altered plasmid and the strain carrying the native pUUH239.2 plasmid (70). Control experiments were conducted for each competition by dye swaps to determine the relative cost of the two different markers, which was then compensated for in the calculation. Nonlinear slopes, as well as outliers, were removed due to the possibility of plasmid loss or medium adaptation mutations (71, 72).

### Relative gene expression of genes carried on pUUH239.2.

Three cultures originating from individual colonies (biological replicates) grown overnight were diluted 1:1,000 in MHB and grown to an OD_600_ of 0.15, i.e., early exponential phase. Aliquots of the cultures were added directly to RNAprotect bacterial reagent (Qiagen), and total RNA was extracted using the RNeasy minikit (Qiagen) according to the manufacturer’s protocol. DNA was removed with the DNase Turbo Free kit (Ambion, Life Technologies). The total RNA concentration was measured with a Qubit 2.0 fluorometer (Invitrogen), and cDNA was produced with the high-capacity reverse transcription kit (Applied Biosystems), using 500 ng of total RNA as the template. The cDNA was diluted and used as the template for real-time quantitative PCR (RT-qPCR) using a MiniOpticon real-time PCR system (Bio-Rad). Chromosomal genes used as references were *cysG* and *hcaT* (73). The primers used are listed in Table S2.

### Transcriptomics and proteomics.

Cultures were grown overnight in biological duplicates and diluted 1:500 in MHB. The cells were grown until an OD_600_ of 0.15 was reached, after which aliquots were taken for RNA extraction and whole-cell proteomics. RNA extraction was performed as described above. For whole-cell proteomics analysis, a 20-mL culture was cooled on ice and centrifuged at 4,500 × *g* at 4°C for 10 min. The supernatant was removed, and the cells were washed twice in 1× PBS. The pellets were stored at −80°C until shipping to the Proteomics Core Facility at the University of Gothenburg for analysis. The full procedure for proteomics analysis is described in Text S1 in the supplemental material. The transcriptomics analysis was performed by BGI, Denmark. Samples were treated according to Solexa sequencing protocols (Illumina Inc., USA), turned into gDNA, and sequenced using the Illumina HiSeq 2000 platform (Illumina Inc., USA). After sequencing, the Illumina pipeline was used for base calling, image analysis, and quality score calibration. The raw reads were quality filtered and aligned to reference sequences using SOAPaligner/SOAP2 (74) and exported as FASTQ files.

10.1128/mbio.03552-21.1 TEXT S1 Supplemental Materials and Methods. Download Text S1, DOCX file, 0.02 MB. Copyright © 2022 Rajer and Sandegren. 2022 Rajer and Sandegren. https://creativecommons.org/licenses/by/4.0/ This content is distributed under the terms of the Creative Commons Attribution 4.0 International license.

### Data availability.

The mass spectrometry proteomics data have been deposited to the ProteomeXchange Consortium via the PRIDE (75) partner repository with the data set identifier PXD014039. Transcriptomic data have been deposited at the NCBI under BioProject accession number PRJNA731092. DNA sequences have been deposited at the NCBI under BioProject accession number PRJNA732311.
