# Supplementary material for: The Role of Antibiotic Resistance Genes in the Fitness Cost of Multiresistance Plasmids
Source: mBio. 2022 Jan 18;13(1):e03552-21. doi: 10.1128/mbio.03552-21 (PMC8764527; doi:10.1128/mbio.03552-21)
Supplement: TABLE S1 [file mbio.03552-21-st001.docx]

**Supplementary Table S1.** Bacterial strains and genotypes.

| **Strain** | **Genotype** | | |
| --- | --- | --- | --- |
|  |  | | |
| DA5438 | *E. coli* MG1655 (*fadK*(ins IS*1*), *yohK*(L34S)) | | |
| DA38186 | *E. coli* F- [*Lambda cI857*, (*attB*-*orf60a*)::*tetAR*(sw), DEL(*cro-bioA*)], *dapA*::scarfree deletion /pUUH239.2 | | |
| DA28426 | DH5-alpha/ pCP20 (AmpR and ZeoR) | | |
| DA28200 | *E. coli galK*::FRTscar-J23101-*SYFP2* | | |
| DA28202 | *E. coli galK*::FRTscar-J23101-*mTagBFP2* | | |
| DA38176 | *E. coli* *galK*::FRTscar-J23101-*SYFP2* /pUUH239.2 | | |
| DA38178 | *E. coli* *galK*::FRTscar-J23101-*mTagBFP2* /pUUH239.2 | | |
| DA38156 | *E. coli* *galK*::FRTscar-J23101-*SYFP2* /pUUH239.2 *mphA*, *mrx*, *mphRA*::FRT | | |
| DA38158 | *E. coli* *galK*::FRTscar-J23101-*mTagBFP2* /pUUH239.2 *mphA*, *mrx*, *mphRA*::FRT | | |
| DA38160 | *E. coli* *galK*::FRTscar-J23101-*SYFP2* /pUUH239.2 *chrA*, *sul1*, *qacEΔ1*, *aadA2*, *dhfr7*, *int*::FRT | | |
| DA38162 | *E. coli* *galK*::FRTscar-J23101-*mTagBFP2* /pUUH239.2 *chrA*, *sul1*, *qacEΔ1*, *aadA2*, *dhfr7*, *int*::FRT | | |
| DA38164 | *E. coli* *galK*::FRTscar-J23101-*SYFP2* /pUUH239.2 *bla*_TEM-1_::FRT | | |
| DA38166 | *E. coli* *galK*::FRTscar-J23101-*mTagBFP2* /pUUH239.2 *bla*_TEM-1_::FRT | | |
| DA38168 | *E. coli* *galK*::FRTscar-J23101-*SYFP2* /pUUH239.2 *aac(6’)-Ib-cr*, *bla*_OXA-1_::FRT | | |
| DA38170 | *E. coli* *galK*::FRTscar-J23101-*mTagBFP2* /pUUH239.2 *aac(6’)-Ib-cr*, *bla*_OXA-1_::FRT | | |
| DA38172 | *E. coli* *galK*::FRTscar-J23101-*SYFP2* /pUUH239.2 *tetR/A*::FRT | | |
| DA38174 | *E. coli* *galK*::FRTscar-J23101-*mTagBFP2* /pUUH239.2 *tetR/A*::FRT | | |
| DA50540 | *E. coli* *galK*::FRTscar-J23101-*SYFP2* /pUUH239.2 *finO*::FRT | | |
| DA50542 | *E. coli* *galK*::FRTscar-J23101-*mTagBFP2* /pUUH239.2 *finO*::FRT | | |
| DA50548 | *E. coli* *galK*::FRTscar-J23101-*SYFP2* /pUUH239.2 *traJ*::FRT | | |
| DA55050 | *E. coli* *galK*::FRTscar-J23101-*mTagBFP2* /pUUH239.2 *traJ*::FRT | | |
| DA51222 | *E. coli galK*::FRTscar-J23101-SYFP2/ pUUH239.2 IS*26*(1-3)::FRT | | |
| DA51224 | *E. coli* *galK*::FRTscar-J23101-*mTagBFP2*/ pUUH239.2 IS*26*(1-3)::FRT | | |
| DA51226 | *E. coli* *galK*::FRTscar-J23101-*SYFP2* /pUUH239.2 IS*26*(1-6)::*cat* | | |
| DA51228 | *E. coli* *galK*::FRTscar-J23101-*mTagBFP2*/ pUUH239.2 IS*26*(1-6)::*cat* | | |
| DA51230 | *E. coli* *galK*::FRTscar-J23101-*SYFP2* /pUUH239.2 IS*26*(3-6)::FRT | | |
| DA51232 | *E. coli* *galK*::FRTscar-J23101-*mTagBFP2* /pUUH239.2 IS*26*(3-6)::FRT | | |
| DA51238 | *E. coli* *galK*::FRTscar-J23101-*SYFP2* /pUUH239.2 IS*26*(3-4)::FRT | | |
| DA51240 | *E. coli* *galK*::FRTscar-J23101-*mTagBFP2* /pUUH239.2 IS*26*(3-4)::FRT | | |
| DA51242 | *E. coli* *galK*::FRTscar-J23101-*SYFP2* /pUUH239.2 IS*26*(1-2)::FRT | | |
| DA51244 | *E. coli* *galK*::FRTscar-J23101-*mTagBFP2* /pUUH239.2 IS*26*(1-2)::FRT | | |
| DA51250 | *E. coli galK*::FRTscar-J23101-SYFP2/ pUUH239.2 IS*26*(1-4)::FRT | | |
| DA51252 | *E. coli galK*::FRTscar-J23101-*mTagBFP2*/ pUUH239.2 IS*26*(1-4)::FRT | | |
| DA57705 | *E. coli* *galK*::FRTscar-J23101-*SYFP2* /pUUH239.2 IS*26*(2-3)::FRT | | |
| DA57706 | *E. coli* *galK*::FRTscar-J23101-*mTagBFP2* /pUUH239.2 IS*26*(2-3)::FRT | | |
| DA50494 | *E. coli* MG1655/ pBAD18-kan | | |
| DA50500 | *E. coli* MG1655/ pBAD18:*bla*_CTX-M-15_ | | |
| DA50506 | *E. coli* MG1655/ pBAD18:*bla*_TEM-1_ | | |
| DA50512 | *E. coli* MG1655/ pBAD18:*bla*_OXA-1_ | | |
| DA57743 | *E. coli* MG1655/ pBAD18:*mphA* | | |
| DA57745 | *E. coli* MG1655/ pBAD18:*mrx* | | |
| DA57747 | *E. coli* MG1655/ pBAD18:*mphR* | | |
| DA57750 | *E. coli* MG1655/ pBAD18:*aac(6’)-Ib-cr* | | |
| DA57752 | *E. coli* MG1655/ pBAD18:*sul-1* | | |
| DA57754 | *E. coli* MG1655/ pBAD18:*aadA2* | | |
| DA57756 | *E. coli* MG1655/ pBAD18:*dhfr* | | |
| DA57758 | *E. coli* MG1655/ pBAD18:*tetR* | | |
| DA57760 | *E. coli* MG1655/ pBAD18:*tetA* | | |
| DA57619 | *E. coli* MG1655/ pBAD18: *bla*_CTX-M-15_ with signal sequence substituted to the signal sequence of *bla*_TEM-1_ | | |
| DA57623 | *E. coli* MG1655/ pBAD18: *bla*_TEM-1_ with signal sequence substituted to the signal sequence of *bla*_CTX-M-15_ | | |
| DA51100 | *E. coli* MG1655/clinical plasmid L | Selection 10 mg/L tmp | Previously published^a^ |
| DA51102 | *E. coli* MG1655/clinical plasmid G | Selection 10 mg/L tmp | Previously published^a^ |
| DA51104 | *E. coli* MG1655/clinical plasmid H | Selection 10 mg/L tmp | Previously published^a^ |
| DA51106 | *E. coli* MG1655/clinical plasmid J | Selection 10 mg/L tmp | Previously published^a^ |
| DA51108 | *E. coli* MG1655/clinical plasmid F | Selection 10 mg/L tmp | Previously published^b^ |
| DA51110 | *E. coli* MG1655/clinical plasmid I | Selection 10 mg/L tmp | Previously published^b^ |
| DA51114 | *E. coli* MG1655/clinical plasmid C | Selection 10 mg/L tmp | Previously published^b^ |
| DA51122 | *E. coli* MG1655/clinical plasmid K | Selection 10 mg/L ctx | Previously published^a^ |
| DA51124 | *E. coli* MG1655/clinical plasmid E | Selection 10 mg/L ctx | Previously published^a^ |
| DA51126 | *E. coli* MG1655/clinical plasmid A | Selection 10 mg/L ctx | Previously published^a^ |
| DA51128 | *E. coli* MG1655/clinical plasmid D | Selection 10 mg/L ctx | Environmental sample |
| DA51138 | *E. coli* MG1655/clinical plasmid B | Selection 15 mg/L tet | Previously published^a^ |
| DA57722 | *E. coli* MG1655/pUUH239.2:FRT_*tetA* | | |
| DA57737 | *E. coli* MG1655/pUUH239.2:FRT_*tetR* | | |
| DA60627 | *E. coli* MG1655/ pBAD18: *bla*_TEM-1_ with substituted signal sequence to ATG | | |
| DA60629 | *E. coli* MG1655/ pBAD18: *bla*_CTX-M-15_ with substituted signal sequence to ATG | | |
| DA60631 | *E. coli* MG1655/ pBAD18:signal sequence of *bla*_TEM-1_ | | |
| DA60633 | *E. coli* MG1655/ pBAD18:signal sequence of *bla*_CTX-M-15_ | | |
| DA60635 | *E. coli* MG1655/ pBAD18:*bla*_CTX-M-14_ | | |
| DA60637 | *E. coli* MG1655/ pBAD18:*bla*_KPC-2_ | | |
| DA60639 | *E. coli* MG1655/ pBAD18:*bla*_NDM-1_ | | |
| DA60641 | *E. coli* MG1655/ pBAD18:*bla*_OXA-48_ | | |
| DA63522 | *E. coli* MG1655/pUUH239.2 | | |
| DA65656 | *E. coli* MG1655/ pBAD18: *ybdM* | | |
| DA65658 | *E. coli* MG1655/ pBAD18: *mmuP* | | |
| DA68693 | *E. coli galK*::FRTscar-J23101-*SYFP2::ybdM* | | |
| DA68695 | *E. coli galK*::FRTscar-J23101-*mTagBFP2::ybdM* | | |
| DA68697 | *E. coli galK*::FRTscar-J23101-*SYFP2::mmuP* | | |
| DA68699 | *E. coli galK*::FRTscar-J23101-*mTagBFP2::mmuP* | | |
| DA68701 | *E. coli galK*::FRTscar-J23101-*SYFP2::ybdM::mmuP* | | |
| DA68703 | *E. coli galK*::FRTscar-J23101-*mTagBFP2::ybdM::mmuP* | | |
| DA68715 | *E. coli galK*::FRTscar-J23101-*SYFP2::ybdM* /pUUH239.2 | | |
| DA68717 | *E. coli galK*::FRTscar-J23101-*mTagBFP2::ybdM* /pUUH239.2 | | |
| DA68719 | *E. coli galK*::FRTscar-J23101-*SYFP2::mmuP* /pUUH239.2 | | |
| DA68721 | *E. coli galK*::FRTscar-J23101-*mTagBFP2::mmuP* /pUUH239.2 | | |
| DA68723 | *E. coli galK*::FRTscar-J23101-*SYFP2::ybdM::mmuP* /pUUH239.2 | | |
| DA68725 | *E. coli galK*::FRTscar-J23101-*mTagBFP2::ybdM::*mmuP /pUUH239.2 | | |
| DA68731 | *E. coli galK*::FRTscar-J23101-*SYFP2::ybdM* /pUUH239.2::*bla*CTX-M-15 | | |
| DA68733 | *E. coli galK*::FRTscar-J23101-*mTagBFP2::ybdM* /pUUH239.2::*bla*CTX-M-15 | | |
| DA68735 | *E. coli galK*::FRTscar-J23101-*SYFP2::mmuP* /pUUH239.2::*bla*CTX-M-15 | | |
| DA68737 | *E. coli galK*::FRTscar-J23101-*mTagBFP2::mmuP* /pUUH239.2::*bla*CTX-M-15 | | |
| DA68739 | *E. coli galK*::FRTscar-J23101-*SYFP2::ybdM::mmuP* /pUUH239.2::*bla*CTX-M-15 | | |
| DA68741 | *E. coli galK*::FRTscar-J23101-*mTagBFP2::ybdM::*mmuP /pUUH239.2::*bla*CTX-M-15 | | |

^a^ doi: 10.1371/journal.pone.0065793

^b^ doi: 10.1128/AAC.02201-18
