# Supplementary material for: The Role of Antibiotic Resistance Genes in the Fitness Cost of Multiresistance Plasmids
Source: mBio. 2022 Jan 18;13(1):e03552-21. doi: 10.1128/mbio.03552-21 (PMC8764527; doi:10.1128/mbio.03552-21)
Supplement: TABLE S3 [file mbio.03552-21-st003.docx]

**Supplementary Table S3.** RPKM data for the two pUUH239.2-containing strains. 50 most expressed transcripts.

| geneID | length | pUUH239.2 A | pUUH239.2 B | Gene |
| --- | --- | --- | --- | --- |
| b1677 | 237 | 28369.1292 | 25689.02272 | *lpp* |
| b0957 | 1041 | 19680.05415 | 20444.37338 | *ompA* |
| b1094 | 237 | 20773.99587 | 20001.78765 | *acpP* |
| b3231 | 429 | 15287.81147 | 17025.5712 | *rplM* |
| b2215 | 1104 | 13596.9747 | 14664.11754 | *ompC* |
| b3307 | 306 | 12184.43219 | 14451.37338 | *rpsN* |
| b3230 | 393 | 14693.2575 | 13806.50836 | *rpsI* |
| b3986 | 366 | 18263.81856 | 13614.64918 | *rplL* |
| lcl\|CP002474.1_143 | 876 | 11055.41237 | 11775.53904 | *ctx-m-15* |
| b3985 | 498 | 15254.57939 | 11626.03349 | *rplJ* |
| b3310 | 372 | 11954.34495 | 11373.78991 | *rplN* |
| b0623 | 210 | 14252.0563 | 11365.34958 | *cspE* |
| b0814 | 516 | 11756.58021 | 11166.74981 | *ompX* |
| b3308 | 540 | 10931.35979 | 11070.71185 | *rplE* |
| b0169 | 726 | 11794.01686 | 10982.76803 | *rpsB* |
| b3340 | 2115 | 12025.43046 | 10709.38419 | *fusA* |
| b3637 | 237 | 10927.63718 | 10302.6379 | *rpmB* |
| b0032 | 1149 | 7675.248285 | 9359.303854 | *carA* |
| b4143 | 1647 | 6752.617083 | 9214.774438 | *groL* |
| b3339 | 1185 | 9497.516953 | 9190.542049 | *tufA* |
| b1779 | 996 | 8344.224339 | 9110.851968 | *gapA* |
| b3295 | 990 | 9541.036063 | 8848.45528 | *rpoA* |
| b3298 | 357 | 8184.057367 | 8819.229129 | *rpsM* |
| b3315 | 333 | 10685.30153 | 8731.813804 | *rplV* |
| b3309 | 315 | 9500.4754 | 8587.737384 | *rplX* |
| b3311 | 255 | 10638.56347 | 8402.506863 | *rpsQ* |
| b3296 | 621 | 7511.717236 | 8373.569498 | *rpsD* |
| b3313 | 411 | 10783.61671 | 8176.447245 | *rplP* |
| b4142 | 294 | 5685.58676 | 7959.952148 | *groS* |
| b3301 | 435 | 8192.102963 | 7938.860537 | *rplO* |
| b3984 | 705 | 8392.761539 | 7892.219064 | *rplA* |
| b3314 | 702 | 10621.32097 | 7583.597463 | *rpsC* |
| b3342 | 375 | 8967.277504 | 7573.898413 | *rpsL* |
| b3312 | 192 | 10513.08262 | 7395.809933 | *rpmC* |
| b3297 | 390 | 5900.742801 | 7301.002181 | *rpsK* |
| b0911 | 1674 | 7834.256048 | 7198.277171 | *rpsA* |
| b3983 | 429 | 7621.879084 | 7194.907736 | *rplK* |
| b4000 | 273 | 6935.577453 | 7057.577455 | *hupA* |
| b1717 | 198 | 8398.071998 | 6898.370198 | *rpmI* |
| b4200 | 396 | 7188.934829 | 6753.61989 | *rpsF* |
| b3980 | 1185 | 6563.128295 | 6740.333547 | *tufB* |
| b3341 | 540 | 7588.015389 | 6700.96313 | *rpsG* |
| b1089 | 174 | 8083.650894 | 6537.220534 | *rpmF* |
| b3318 | 303 | 8219.671415 | 6504.609815 | *rplW* |
| b3320 | 630 | 7985.790058 | 6484.893511 | *rplC* |
| b4201 | 315 | 6258.878628 | 6390.401403 | *priB* |
| b3317 | 822 | 8623.118677 | 6379.090706 | *rplB* |
| b3186 | 312 | 6850.658604 | 6332.906454 | *rplU* |
| b3304 | 354 | 6196.83327 | 6281.343704 | *rplR* |
