# Supplementary material for: The Role of Antibiotic Resistance Genes in the Fitness Cost of Multiresistance Plasmids
Source: mBio. 2022 Jan 18;13(1):e03552-21. doi: 10.1128/mbio.03552-21 (PMC8764527; doi:10.1128/mbio.03552-21)
Supplement: TABLE S4 [file mbio.03552-21-st004.docx]

**Supplementary Table S4.** Susceptibility testing (mg/L) of pBAD18 expression vectors containing cloned resistance genes. Tryptone broth agar with addition of 50ug/ml kanamycin and 0.05% L-arabinose was used.

| Strain / antibiotic | TC | SM | TR | EM | SX | CIP | AM | CT | IMI |
| --- | --- | --- | --- | --- | --- | --- | --- | --- | --- |
|  |  |  |  |  |  |  |  |  |  |
| empty pBAD18 | 3 | 3 | 6 | 48 | 4 | 0.008 | 4 | 0.023 | 0.38 |
| *mphA* |  |  |  | >256 |  |  |  |  |  |
| *aac(6’)-Ib-cr* |  |  |  |  |  | 0.032 |  |  |  |
| *sul1* |  |  |  |  | >1024 |  |  |  |  |
| *aadA2* |  | 256 |  |  |  |  |  |  |  |
| *dhfrXII* |  |  | >32 |  |  |  |  |  |  |
| *tetA* | 48 |  |  |  |  |  |  |  |  |
| *bla*_TEM-1_ |  |  |  |  |  |  | >256 |  |  |
| *bla*_OXA-1_ |  |  |  |  |  |  | >256 |  |  |
| *bla*_CTX-M-15_ |  |  |  |  |  |  |  | >32 |  |
| *bla*_TEM-1_with SS of *bla*_CTX-M-15_ |  |  |  |  |  |  | >256 |  |  |
| *bla*_CTX-M-15_with SS of *bla*_TEM-1_ |  |  |  |  |  |  |  | >32 |  |
| *bla*_TEM-1_with no SS |  |  |  |  |  |  | 2 |  |  |
| *bla*_CTX-M-15_with no SS |  |  |  |  |  |  |  | 6 |  |
| *bla*_CTX-M-14_ |  |  |  |  |  |  |  | >32 |  |
| *bla*_KPC-2_ |  |  |  |  |  |  |  |  | 3 |
| *bla*_NDM-1_ |  |  |  |  |  |  |  |  | 8 |
| *bla*_OXA-48_ |  |  |  |  |  |  |  |  | 2 |

TC tetracycline, SM streptomycin, TR trimethoprim, EM erythromycin, SX sulfamethoxazole, CIP ciprofloxacin, AM ampicillin, CT cefotaxime, IMI imipenem

SS signal sequence
