# Supplementary material for: The Role of Antibiotic Resistance Genes in the Fitness Cost of Multiresistance Plasmids
Source: mBio. 2022 Jan 18;13(1):e03552-21. doi: 10.1128/mbio.03552-21 (PMC8764527; doi:10.1128/mbio.03552-21)
Supplement: TABLE S5 [file mbio.03552-21-st005.docx]

**Supplementary Table S5.** Transcriptomics data of a plasmid-containing strains and its significant up- and down-regulated chromosomal genes compared to plasmid-free cells. Values are in a log2-scale.

|  | pUUH239.2* | | ΔResCass* | | Δ*tetRA** | | Δ*bla*_CTX-M-15_* | | *tetA*:FRT* | | *tetR*:FRT* | |
| --- | --- | --- | --- | --- | --- | --- | --- | --- | --- | --- | --- | --- |
| Gene | A | B | A | B | A | B | A | B | A | B | A | B |
| *insO* | 5.10 | 5.17 | 5.06 | 3.97 | 4.79 | 5.39 | 6.19 | 7.06 | 6.19 | 4.66 | 6.06 | 5.17 |
| *ybcW* | 2.89 | 2.57 | 2.66 | 2.87 | 3.16 | 3.36 | 2.87 | 2.64 | 3.01 | 3.36 | 3.23 | 2.31 |
| *cydA* | 2.83 | 2.77 | 3.29 | 3.27 | 2.83 | 2.95 | 2.97 | 3.05 | 3.05 | 2.60 | 2.93 | 3.03 |
| *adiY* | 2.73 | 2.87 | 2.97 | 2.85 | 3.76 | 3.71 | 3.89 | 3.58 | 3.84 | 2.85 | 3.81 | 3.89 |
| *ybgE* | 2.71 | 2.68 | 3.12 | 3.32 | 2.69 | 2.73 | 2.85 | 2.68 | 2.85 | 2.38 | 2.71 | 2.83 |
| *cydB* | 2.64 | 2.68 | 3.23 | 3.18 | 2.69 | 2.77 | 2.91 | 2.95 | 2.99 | 2.50 | 2.77 | 2.91 |
| *cydX* | 2.62 | 2.27 | 3.68 | 3.51 | 2.93 | 2.95 | 2.57 | 2.35 | 2.51 | 2.30 | 2.57 | 2.60 |
| *focA* | 2.20 | 2.73 | 2.45 | 2.50 | 2.51 | 2.51 | 2.53 | 2.55 | 2.58 | 2.43 | 2.30 | 2.48 |
| *ymgD* | 2.06 | 2.22 | 1.12 | 0.98 | 2.57 | 2.46 | 2.93 | 3.05 | 2.46 | 2.31 | 2.11 | 2.50 |
| *yeeE* | 1.90 | 1.43 | 2.38 | 2.53 | 1.48 | 0.90 | 1.31 | 0.60 | 1.14 | 1.92 | 1.36 | 1.71 |
| *InsH1* | 1.86 | 2.19 | 2.48 | 2.22 | 2.38 | 2.73 | 2.79 | 2.66 | 2.68 | 2.64 | 2.45 | 2.33 |
| *ymgG* | 1.82 | 2.33 | 1.22 | 0.99 | 2.62 | 2.45 | 3.14 | 3.16 | 2.79 | 2.48 | 2.31 | 2.50 |
| *lldP* | 0.64 | 0.45 | 0.44 | 0.46 | 0.42 | 0.56 | 0.44 | 0.49 | 0.54 | 0.41 | 0.46 | 0.52 |
| *entA* | 0.59 | 0.72 | 0.44 | 0.49 | 0.59 | 0.39 | 0.44 | 0.51 | 0.40 | 0.50 | 0.54 | 0.45 |
| *entH* | 0.56 | 0.67 | 0.61 | 0.64 | 0.61 | 0.39 | 0.47 | 0.43 | 0.55 | 0.60 | 0.38 | 0.49 |
| *entF* | 0.52 | 0.57 | 0.51 | 0.47 | 0.62 | 0.44 | 0.47 | 0.45 | 0.48 | 0.58 | 0.54 | 0.47 |
| *entE* | 0.49 | 0.70 | 0.50 | 0.45 | 0.60 | 0.35 | 0.40 | 0.40 | 0.39 | 0.56 | 0.54 | 0.44 |
| *srlD* | 0.49 | 0.41 | 0.81 | 0.94 | 0.56 | 0.39 | 0.55 | 0.60 | 0.53 | 0.52 | 0.49 | 0.42 |
| *sucA* | 0.48 | 0.57 | 0.53 | 0.48 | 0.50 | 0.45 | 0.48 | 0.57 | 0.47 | 0.44 | 0.46 | 0.47 |
| *glcC* | 0.48 | 0.55 | 0.34 | 0.48 | 0.39 | 0.48 | 0.43 | 0.37 | 0.55 | 0.58 | 0.63 | 0.46 |
| *glnL* | 0.48 | 0.72 | 0.50 | 0.41 | 0.70 | 1.06 | 0.91 | 1.34 | 0.97 | 0.48 | 0.78 | 0.74 |
| *mdh* | 0.48 | 0.47 | 0.51 | 0.54 | 0.53 | 0.51 | 0.52 | 0.52 | 0.52 | 0.53 | 0.52 | 0.50 |
| *pqqL* | 0.47 | 0.46 | 1.29 | 1.37 | 0.36 | 0.44 | 0.39 | 0.30 | 0.40 | 0.46 | 0.38 | 0.37 |
| *entC* | 0.46 | 0.63 | 0.46 | 0.44 | 0.64 | 0.41 | 0.43 | 0.38 | 0.37 | 0.58 | 0.59 | 0.42 |
| *sucB* | 0.45 | 0.47 | 0.47 | 0.44 | 0.43 | 0.43 | 0.46 | 0.49 | 0.43 | 0.42 | 0.43 | 0.47 |
| *yddA* | 0.45 | 0.48 | 1.34 | 1.54 | 0.56 | 0.19 | 0.30 | 0.27 | 0.48 | 0.87 | 0.40 | 0.29 |
| *nrdF* | 0.44 | 0.51 | 1.06 | 1.07 | 0.46 | 0.44 | 0.40 | 0.46 | 0.57 | 0.57 | 0.52 | 0.47 |
| *glnA* | 0.44 | 0.69 | 0.41 | 0.38 | 0.74 | 1.14 | 0.50 | 1.33 | 0.91 | 0.48 | 0.83 | 0.68 |
| *gcvT* | 0.43 | 0.49 | 0.81 | 0.85 | 0.55 | 0.51 | 0.50 | 0.57 | 0.52 | 0.44 | 0.45 | 0.53 |
| *yddB* | 0.43 | 0.41 | 1.15 | 1.31 | 0.51 | 0.37 | 0.30 | 0.35 | 0.34 | 0.47 | 0.47 | 0.36 |
| *sucD* | 0.43 | 0.45 | 0.41 | 0.42 | 0.42 | 0.43 | 0.44 | 0.50 | 0.45 | 0.41 | 0.42 | 0.43 |
| *cirA* | 0.42 | 0.62 | 0.47 | 0.48 | 0.58 | 0.39 | 0.42 | 0.38 | 0.40 | 0.57 | 0.53 | 0.40 |
| *ndk* | 0.42 | 0.35 | 0.37 | 0.40 | 0.39 | 0.35 | 0.40 | 0.38 | 0.40 | 0.44 | 0.41 | 0.38 |
| *efeB* | 0.42 | 0.52 | 0.43 | 0.55 | 0.45 | 0.38 | 0.42 | 0.44 | 0.45 | 0.41 | 0.50 | 0.43 |
| *lldR* | 0.42 | 0.31 | 0.51 | 0.32 | 0.34 | 0.36 | 0.40 | 0.33 | 0.46 | 0.36 | 0.32 | 0.30 |
| *mhpR* | 0.42 | 0.51 | 0.47 | 0.54 | 0.48 | 0.66 | 0.41 | 0.53 | 0.57 | 0.38 | 0.38 | 0.51 |
| *msrB* | 0.42 | 0.54 | 0.60 | 0.56 | 0.48 | 0.53 | 0.47 | 0.48 | 0.46 | 0.41 | 0.46 | 0.49 |
| *fes* | 0.41 | 0.65 | 0.31 | 0.45 | 0.66 | 0.34 | 0.37 | 0.34 | 0.32 | 0.51 | 0.45 | 0.31 |
| *lldD* | 0.40 | 0.36 | 0.35 | 0.32 | 0.35 | 0.31 | 0.37 | 0.39 | 0.37 | 0.38 | 0.32 | 0.32 |
| *ylaC* | 0.40 | 0.35 | 0.54 | 0.60 | 0.41 | 0.44 | 0.40 | 0.38 | 0.41 | 0.36 | 0.38 | 0.42 |
| *putA* | 0.40 | 0.37 | 0.41 | 0.33 | 0.35 | 0.39 | 0.39 | 0.48 | 0.37 | 0.41 | 0.37 | 0.42 |
| *argT* | 0.39 | 0.31 | 0.38 | 0.35 | 0.37 | 0.51 | 0.37 | 0.54 | 0.36 | 0.37 | 0.34 | 0.38 |
| *mglB* | 0.39 | 0.35 | 0.34 | 0.39 | 0.48 | 0.39 | 0.43 | 0.32 | 0.31 | 0.50 | 0.40 | 0.36 |
| *ompT* | 0.38 | 0.40 | 1.22 | 1.34 | 0.39 | 0.47 | 0.37 | 0.37 | 0.38 | 0.40 | 0.41 | 0.43 |
| *sthA* | 0.37 | 0.31 | 0.33 | 0.32 | 0.34 | 0.36 | 0.37 | 0.36 | 0.35 | 0.39 | 0.33 | 0.35 |
| *dctA* | 0.37 | 0.47 | 0.45 | 0.38 | 0.51 | 0.36 | 0.46 | 0.51 | 0.41 | 0.51 | 0.45 | 0.43 |
| *icd* | 0.37 | 0.48 | 0.50 | 0.49 | 0.48 | 0.44 | 0.47 | 0.50 | 0.47 | 0.46 | 0.46 | 0.44 |
| *sdhC* | 0.37 | 0.26 | 0.31 | 0.30 | 0.27 | 0.25 | 0.17 | 0.26 | 0.28 | 0.22 | 0.29 | 0.27 |
| *nrdE* | 0.37 | 0.51 | 0.66 | 0.84 | 0.49 | 0.36 | 0.49 | 0.50 | 0.47 | 0.46 | 0.54 | 0.39 |
| *efeO* | 0.36 | 0.50 | 0.49 | 0.49 | 0.47 | 0.41 | 0.42 | 0.46 | 0.40 | 0.49 | 0.49 | 0.38 |
| *cyoE* | 0.36 | 0.36 | 0.46 | 0.50 | 0.33 | 0.25 | 0.29 | 0.31 | 0.28 | 0.37 | 0.32 | 0.30 |
| *osmY* | 0.36 | 0.47 | 0.46 | 0.43 | 0.47 | 0.54 | 0.56 | 0.55 | 0.42 | 0.46 | 1.71 | 0.52 |
| *cyoD* | 0.36 | 0.32 | 0.45 | 0.46 | 0.29 | 0.23 | 0.25 | 0.28 | 0.26 | 0.33 | 0.28 | 0.26 |
| *entB* | 0.35 | 0.75 | 0.60 | 0.64 | 0.62 | 0.34 | 0.40 | 0.41 | 0.35 | 0.49 | 0.55 | 0.39 |
| *aldA* | 0.35 | 0.44 | 0.44 | 0.41 | 0.43 | 0.43 | 0.46 | 0.48 | 0.40 | 0.50 | 0.44 | 0.45 |
| *cyoB* | 0.35 | 0.29 | 0.40 | 0.41 | 0.28 | 0.24 | 0.26 | 0.28 | 0.27 | 0.32 | 0.27 | 0.27 |
| *sdhB* | 0.35 | 0.33 | 0.39 | 0.35 | 0.35 | 0.31 | 0.33 | 0.36 | 0.30 | 0.40 | 0.33 | 0.32 |
| *astC* | 0.35 | 0.16 | 0.25 | 0.26 | 0.26 | 0.96 | 0.50 | 0.99 | 0.29 | 0.11 | 0.40 | 0.30 |
| *sucC* | 0.35 | 0.46 | 0.42 | 0.40 | 0.44 | 0.42 | 0.44 | 0.48 | 0.42 | 0.40 | 0.43 | 0.42 |
| *yncD* | 0.34 | 0.38 | 0.39 | 0.43 | 0.35 | 0.33 | 0.33 | 0.30 | 0.31 | 0.36 | 0.36 | 0.36 |
| *sdhD* | 0.33 | 0.30 | 0.13 | 0.25 | 0.22 | 0.18 | 0.15 | 0.29 | 0.24 | 0.26 | 0.20 | 0.26 |
| *yejG* | 0.33 | 0.31 | 0.31 | 0.34 | 0.35 | 0.33 | 0.32 | 0.29 | 0.35 | 0.30 | 0.34 | 0.31 |
| *yqeF* | 0.33 | 0.33 | 0.31 | 0.38 | 0.46 | 0.37 | 0.46 | 0.34 | 0.35 | 0.42 | 0.38 | 0.41 |
| *efeU* | 0.32 | 0.56 | 0.56 | 0.49 | 0.47 | 0.39 | 0.50 | 0.45 | 0.41 | 0.45 | 0.45 | 0.43 |
| *gcd* | 0.32 | 0.38 | 0.43 | 0.39 | 0.34 | 0.34 | 0.34 | 0.36 | 0.35 | 0.31 | 0.35 | 0.34 |
| *yigI* | 0.32 | 0.26 | 0.31 | 0.29 | 0.28 | 0.29 | 0.30 | 0.35 | 0.35 | 0.28 | 0.26 | 0.28 |
| *cyoC* | 0.32 | 0.32 | 0.44 | 0.47 | 0.31 | 0.23 | 0.28 | 0.27 | 0.26 | 0.32 | 0.29 | 0.28 |
| *fumC* | 0.31 | 0.33 | 0.37 | 0.35 | 0.33 | 0.41 | 0.48 | 0.36 | 0.34 | 0.34 | 0.30 | 0.37 |
| *sdhA* | 0.30 | 0.25 | 0.31 | 0.30 | 0.28 | 0.28 | 0.28 | 0.31 | 0.26 | 0.31 | 0.27 | 0.25 |
| *fumA* | 0.29 | 0.33 | 0.33 | 0.40 | 0.30 | 0.33 | 0.34 | 0.34 | 0.32 | 0.29 | 0.34 | 0.34 |
| *cyoA* | 0.28 | 0.24 | 0.32 | 0.34 | 0.25 | 0.23 | 0.23 | 0.25 | 0.23 | 0.29 | 0.25 | 0.22 |
| *ugpB* | 0.26 | 0.36 | 0.41 | 0.33 | 0.29 | 0.56 | 0.44 | 0.45 | 0.32 | 0.33 | 0.46 | 0.41 |
| *dadA* | 0.26 | 0.27 | 0.43 | 0.36 | 0.31 | 0.27 | 0.30 | 0.29 | 0.24 | 0.29 | 0.28 | 0.29 |
| *mntS* | 0.26 | 0.29 | 0.23 | 0.49 | 0.53 | 0.54 | 0.38 | 0.42 | 0.38 | 0.42 | 0.44 | 0.47 |
| *dadX* | 0.24 | 0.30 | 0.44 | 0.39 | 0.30 | 0.31 | 0.31 | 0.37 | 0.32 | 0.37 | 0.33 | 0.31 |
| *ydcI* | 0.20 | 0.19 | 0.25 | 0.22 | 0.20 | 0.23 | 0.24 | 0.22 | 0.21 | 0.21 | 0.19 | 0.18 |
| *phoH* | 0.19 | 0.32 | 0.30 | 0.28 | 0.32 | 0.32 | 0.25 | 0.27 | 0.23 | 0.29 | 0.27 | 0.28 |
| *mqo* | 0.19 | 0.25 | 0.25 | 0.22 | 0.22 | 0.17 | 0.17 | 0.20 | 0.19 | 0.22 | 0.21 | 0.20 |
| *ynaJ* | 0.18 | 0.19 | 0.14 | 0.21 | 0.17 | 0.16 | 0.15 | 0.15 | 0.20 | 0.19 | 0.20 | 0.20 |
| *glnK* | 0.05 | 0.16 | 0.04 | 0.06 | 0.18 | 0.94 | 0.32 | 1.21 | 0.42 | 0.06 | 0.21 | 0.09 |

* All comparisons are made against the parental *E. coli* K-12 MG1655 not containing a plasmid.

Empty boxes indicate no significant up- or down regulation in that specific replicate of the specific gene.

Heat map colors indicate up regulation (green) and down regulation (yellow, red).
