# Supplementary material for: The Role of Antibiotic Resistance Genes in the Fitness Cost of Multiresistance Plasmids
Source: mBio. 2022 Jan 18;13(1):e03552-21. doi: 10.1128/mbio.03552-21 (PMC8764527; doi:10.1128/mbio.03552-21)
Supplement: TABLE S6 [file mbio.03552-21-st006.docx]

**Supplementary Table S6.** Enriched KEGG pathways of a strain carrying pUUH239.2 compared to a strain without plasmid.

| **KEGG ID** | **RNAseq** | **Pathway** | **FDR*** | **Genes matching KEGG network** |
| --- | --- | --- | --- | --- |
| eco00190 | + | Oxidative phosphorylation | 0.00044 | *cydA, cydB, cydX* |
| eco02020 | + | Two-component system | 0.0075 | *cydA, cydB, cydX* |
| eco00020 | - | Citrate cycle (TCA cycle) | 4.95E-13 | *fumA, fumC, icd, mdh, mqo, sdhA, sdhB, sdhC, sdhD, sucA, sucB, sucC, sucD* |
| eco01130 | - | Biosynthesis of antibiotics | 5.58E-12 | *entA, entB, entC, entE, entF, fumA, fumC, gcd, gcvT, icd, mdh, mqo, ndk, putA, sdhA, sdhB, sdhC, sdhD, sucA, sucB, sucC, sucD, yqeF* |
| eco01110 | - | Biosynthesis of secondary metabolites | 6.36E-10 | *cyoE, entA, entB, entC, entE, entF, fumA, fumC, gcd, gcvT, icd, mdh, mqo, ndk, putA, sdhA, sdhB, sdhC, sdhD, sucA, sucB, sucC, sucD, yqeF* |
| eco01200 | - | Carbon metabolism | 4.55E-09 | *fumA, fumC, gcvT, icd, mdh, mqo, sdhA, sdhB, sdhC, sdhD, sucA, sucB, sucC, sucD, yqeF* |
| eco01100 | - | Metabolic pathways | 4.01E-07 | *astC, cyoA, cyoB, cyoC, cyoD, cyoE, dadX, entC, fumA, fumC, gcd, gcvT, glnA, icd, lldD, mdh, mqo, ndk, nrdE, nrdF, putA, sdhA, sdhB, sdhC, sdhD, sthA, sucA, sucB, sucC, sucD, yqeF* |
| eco00190 | - | Oxidative phosphorylation | 6.12E-07 | *cyoA, cyoB, cyoC, cyoD, cyoE, sdhA, sdhB, sdhC, sdhD* |
| eco01053 | - | Biosynthesis of siderophore group non-ribosomal peptides | 4.09E-06 | *entA, entB, entC, entE, entF* |
| eco01120 | - | Microbial metabolism in diverse environments | 5.29E-05 | *aldA, fumA, fumC, glnA, icd, mdh, mqo, sdhA, sdhB, sdhC, sdhD, sucA, sucB, sucC, sucD, yqeF* |
| eco00620 | - | Pyruvate metabolism | 0.00017 | *aldA, fumA, fumC, lldD, mdh, mqo, yqeF* |
| eco00650 | - | Butanoate metabolism | 0.0021 | *sdhA, sdhB, sdhC, sdhD, yqeF* |
| eco00630 | - | Glyoxylate and dicarboxylate metabolism | 0.003 | *aldA, gcvT, glnA, mdh, yqeF* |
| eco00310 | - | Lysine degradation | 0.0057 | *sucA, sucB, yqeF* |
| eco00380 | - | Tryptophan metabolism | 0.0426 | *sucA, yqeF* |
| eco00660 | - | C5-Branched dibasic acid metabolism | 0.047 | *sucC, sucD* |

Table done at https://string-db.org/

* FDR = false discovery rate, Szklarczyk et al. Nucleic Acids Res. 2015 43(Database issue):D447-52

+ up regulated pathways, - down regulated pathways
