# Supplementary material for: The Role of Antibiotic Resistance Genes in the Fitness Cost of Multiresistance Plasmids
Source: mBio. 2022 Jan 18;13(1):e03552-21. doi: 10.1128/mbio.03552-21 (PMC8764527; doi:10.1128/mbio.03552-21)
Supplement: TABLE S2 [file mbio.03552-21-st002.docx]

**Supplementary Table S2.** Primer sequences for deletions on the pUUH239.2 plasmid and chromosome, as well as for cloning of resistance genes.

| **Primer** | **Sequence (5’ 🡪 3’)** |
| --- | --- |
|  |  |
| Del_dapA_Fwd2 | GTTCGACAAATAGTTTGTTGTGTAATGGCATCAGACGCTGGTGTAGGCTGGAGCTGCTTC |
| Del_dapA_Rev2 | AACGTACCATTGAGACACTTGTTTGCACAGAGGATGGCCCCATATGAATATCCTCCTTAG |
| dapA_repair | GTTCGACAAATAGTTTGTTGTGTAATGGCATCAGACGCTGGGGCCATCCTCTGTGCAAACAAGTGCTCAATGGTACGTT |
| DelIntP1 | CAAACCGCCGTAGAGGTCTTTGTCAGTCATTTTTTGTGCCGTGTAGGCTGGAGCTGCTTC |
| DelIntP2 | CGCAGGGGTAGTGAATCCGCCAGGATTGACTTGCGCTGCCCATATGAATATCCTCCTTAG |
| DelTEM1P3 | GGTCTGACAGTTACCAATGCTTAATCAGTGAGGCACCTATGTGTAGGCTGGAGCTGCTTC |
| DelTEM1P4 | AAGGAAGAGTATGAGTATTCAACATTTTCGTGTCGCCCTTCATATGAATATCCTCCTTAG |
| DelCTXM15P1 | ATGTTGTTGTTATTTCGTATCTTCCAGAATAAGGAATCCCGTGTAGGCTGGAGCTGCTTC |
| DelCTXM15P2 | AAACAAAAACGGAATGAGTTTCCCCATTCCGTTTCCGCTACATATGAATATCCTCCTTAG |
| DelCTXM15P3 | AAGGAATCCCATGGTTAAAAAATCACTGCGCCAGTTCACGGTGTAGGCTGGAGCTGCTTC |
| DelCTXM15P4 | GTTTCCGCTATTACAAACCGTCGGTGACGATTTTAGCCGCCATATGAATATCCTCCTTAG |
| DelCTXM15P5 | GAAGCTAATAAAAAACACACGTGGAATTTAGGGACTATTCGTGTAGGCTGGAGCTGCTTC |
| DelCTXM15P6 | AGCGCAGGTGGGCGACAGCACTTTTGCCGTCTAAGGCGATCATATGAATATCCTCCTTAG |
| DelAAC(6)P1 | CGTGCATAATAAGCCCTACACAAATTGGGAGTTAGACATCGTGTAGGCTGGAGCTGCTTC |
| DelOXA1P2 | GACTTGATTGAAGGGTTGGGCGATTTTGCCATTAGATTTTCATATGAATATCCTCCTTAG |
| Del_IS26_P1^#^ | ATGGAGCTGCACATGAACCCATTCAAAGGCCGGCATTTTCGTGTGTAGGCTGGAGCTGCTTC |
| Del_IS26_P2^#^ | TTACATTTCAAAAACTCTGCTTACCAGGCGCATTTCGCCCCATATGAATATCCTCCTTAG |
| Del_IS26_P3^#^ | AGCGTGACATCATTCTGTGGGCCGTACGCTGGTACTGCAACATATGAATATCCTCCTTAG |
| Del_IS26_P7^#^ | TTACATTTCAAAAACTCTGCTTACCAGGCGCATTTCGCCCGTGTAGGCTGGAGCTGCTTC |
| Del_IS26_P8^#^ | AGGGGATCACCATAATAAAATGCTGAGGCCTGGCCTTTGCCATATGAATATCCTCCTTAGTTCC |
| Del_finO_P3 | ACGCAGGAGCAGGAAAGGGCGATGTCGTTCAGGTATAGTAGTGTAGGCTGGAGCTGCTTC |
| Del_finO_P4 | TTCAGGCATACCCTCAGCTAATTTTCGTTACATCTATTAGCATATGAATATCCTCCTTAG |
| aadA2_XbaI | TCCTCTAGAAGGAGGGACATCATGAGGGAAGCGGTGACCATCGA |
| aadA2_PstI | ATGCCTGCAGAATTGTTAGACATCATTTACCAACTGACTTGATG |
| dhfr_XbaI | TCCTCTAGAAGGAGGAGCCATATGAACTCGGAATCAGTACGCAT |
| dhfr_PstI | ATGCCTGCAGTTGACGGAATGGTTAGCCGTTTCGACGCGCATAA |
| sul1_EcoRI | AGCGAATTCAGGAGGGACGCCATGGTGACGGTGTTCGGCAT |
| sul1_XbaI | GACTCTAGAGGAAGGTGAATGCTAGGCATGATCTAACCCTC |
| mphA_EcoRI | AGCGAATTCAGGAGGTGAATCATGACCGTAGTCACGACCGC |
| mphA_XbaI | GACTCTAGATCGATGGAAGGGTCATTCCGCTGCGGCGAGCT |
| mrx_EcoRI | AGCGAATTCAGGAGGAGCGGAATGAGCGAACGTCGATATAG |
| mrx_XbaI | GACTCTAGATCGATGGAAGGGTCAGTGTTCACCTTCTGTAT |
| mphR_EcoRI | AGCGAATTCAGGAGGACACTGATGCCCCGCCCCAAGCTCAA |
| mphR_XbaI | GACTCTAGAGCACACCTCCGTTTACGCATGTGCCTGGAGGA |
| Cl_aac(6)_EcoRI | AGCGAATTCAGGAGGAGGCCTTTGAATAAGACAAAAGGCTG |
| Cl_aac(6)_XbaI | GACTCTAGATCGATGGAAGGGTTAGGCATCACTGCGTGTTC |
| Cl_tetA_EcoRI | AGCGAATTCAGGAGGCCAGACGTGAAACCCAACAGACCCCT |
| Cl_tetA_XbaI | GACTCTAGATCGTTTCCACGATCAGCGATCGGCTCGTTGCC |
| Cl_tetR_EcoRI | AGCGAATTCAGGAGGCATATTATGTTTATCAGTGATAAAGT |
| Cl_tetR_XbaI | GACTCTAGACAGCGGTCCTGATCAATCGTCACCCTTTCTCG |
| Cl_ndm1_EcoRI  Cl_ndm1_XbaI | GCGAATTCAGGAGGACACTGATGGAATTGCCCAATATTAT  ACTCTAGAGCACACCTCCGTTCAGCGCAGCTTGTCGGCCA |
| Cl_oxa48_EcoRI  Cl_oxa48_XbaI | GCGAATTCAGGAGGACACTGATGCGTGTATTAGCCTTATC  ACTCTAGAGCACACCTCCGTCTAGGGAATAATTTTTTCCT |
| Cl_kpc2_EcoRI  Cl_kpc2_XbaI | GCGAATTCAGGAGGACACTGATGTCACTGTATCGCCGTCT  ACTCTAGAGCACACCTCCGTTTACTGCCCGTTGACGCCCA |
| Cl_ctxm14_EcoRI  Cl_ctxm14_XbaI | GCGAATTCAGGAGGACACTGATGGTGACAAAGAGAGTGCA  ACTCTAGAGCACACCTCCGTTTACAGCCCTTCGGCGATGA |
| Cl_ctxm15_noSS_EcoRI  Cl_tem1_noSS_EcoRI | GCGAATTCAGGAGGAATCCC*ATG*CAAACGGCGGACGTACAGCA  GCGAATTCAGGAGGAAGAGT*ATG*CACCCAGAAACGCTGGTGAA |
| Cl_SS_tem1_EcoRI  Cl_SS_tem1_XbaI | GCGAATTCAGGAGGAAGAGTATGAGTATTCAACATTTTCG  ACTCTAGATTGGTCTGACAGTTAAGCAAAAACAGGAAGGC |
| Cl_SS_ctxm15_EcoRI | GCGAATTCAGGAGGAATCCCATGGTTAAAAAATCACTGCG |
| Cl_SS_ctxm15_XbaI | ACTCTAGACCGTTTCCGCTATTACGCATACAGCGGCACAC |
| *bla*_TEM-1_ with *bla*_CTX-M-15_ signal sequence | GCGAATTCAGGAGGAAGAGT**ATGGTTAAAAAATCACTGCGCCAGTTCACGCTGATGGCGACGGCAACCGTCACGCTGTTGTTAGGAAGTGTGCCGCTGTATGCG**CACCCAGAAACGCTGGTGAAAGTAAAAGATGCTGAAGATCAGTTGGGTGCACGAGTGGGTTACATCGAACTGGATCTCAACAGCGGTAAGATCCTTGAGAGTTTTCGCCCCGAAGAACGTTTTCCAATGATGAGCACTTTTAAAGTTCTGCTATGTGGTGCGGTATTATCCCGTGTTGACGCCGGGCAAGAGCAACTCGGTCGCCGCATACACTATTCTCAGAATGACTTGGTTGAGTACTCACCAGTCACAGAAAAGCATCTTACGGATGGCATGACAGTAAGAGAATTATGCAGTGCTGCCATAACCATGAGTGATAACACTGCTGCCAACTTACTTCTGACAACGATCGGAGGACCGAAGGAGCTAACCGCTTTTTTGCACAACATGGGGGATCATGTAACTCGCCTTGATCGTTGGGAACCGGAGCTGAATGAAGCCATACCAAACGACGAGCGTGACACCACGATGCCTGCAGCAATGGCAACAACGTTGCGCAAACTATTAACTGGCGAACTACTTACTCTAGCTTCCCGGCAACAATTAATAGACTGGATGGAGGCGGATAAAGTTGCAGGACCACTTCTGCGCTCGGCCCTTCCGGCTGGCTGGTTTATTGCTGATAAATCTGGAGCCGGTGAGCGTGGGTCTCGCGGTATCATTGCAGCACTGGGGCCAGATGGTAAGCCCTCCCGTATCGTAGTTATCTACACGACGGGGAGTCAGGCAACTATGGATGAACGAAATAGACAGATCGCTGAGATAGGTGCCTCACTGATTAAGCATTGGTAACTGTCAGACCAATCTAGAG |
| *bla*_CTX-M-15_ with *bla*_TEM-1_ signal sequence | GCGAATTCAGGAGGAATCCC**ATGAGTATTCAACATTTTCGTGTCGCCCTTATTCCCTTTTTTGCGGCATTTTGCCTTCCTGTTTTTGCT**CAAACGGCGGACGTACAGCAAAAACTTGCCGAATTAGAGCGGCAGTCGGGAGGCAGACTGGGTGTGGCATTGATTAACACAGCAGATAATTCGCAAATACTTTATCGTGCTGATGAGCGCTTTGCGATGTGCAGCACCAGTAAAGTGATGGCCGCGGCCGCGGTGCTGAAGAAAAGTGAAAGCGAACCGAATCTGTTAAATCAGCGAGTTGAGATCAAAAAATCTGACCTTGTTAACTATAATCCGATTGCGGAAAAGCACGTCAATGGGACGATGTCACTGGCTGAGCTTAGCGCGGCCGCGCTACAGTACAGCGATAACGTGGCGATGAATAAGCTGATTGCTCACGTTGGCGGCCCGGCTAGCGTCACCGCGTTCGCCCGACAGCTGGGAGACGAAACGTTCCGTCTCGACCGTACCGAGCCGACGTTAAACACCGCCATTCCGGGCGATCCGCGTGATACCACTTCACCTCGGGCAATGGCGCAAACTCTGCGGAATCTGACGCTGGGTAAAGCATTGGGCGACAGCCAACGGGCGCAGCTGGTGACATGGATGAAAGGCAATACCACCGGTGCAGCGAGCATTCAGGCTGGACTGCCTGCTTCCTGGGTTGTGGGGGATAAAACCGGCAGCGGTGGCTATGGCACCACCAACGATATCGCGGTGATCTGGCCAAAAGATCGTGCGCCGCTGATTCTGGTCACTTACTTCACCCAGCCTCAACCTAAGGCAGAAAGCCGTCGCGATGTATTAGCGTCGGCGGCTAAAATCGTCACCGACGGTTTGTAATAGCGGAAACGGTCTAGAG |
| qPCR_*cysG*_P1 | TTGTCGGCGGTGGTGATGTC |
| qPCR*_cysG*_P2 | ATGCGGTGAACTGTGGAATAAACG |
| qPCR_*hcaT*_P1 | TTGTCGGCGGTGGTGATGTC |
| qPCR_*hcaT*_P2 | CCAACCACGCAGACCAACC |
| qPCR_*mph(A)_*P1 | CGCGCACCTTATGGTCTTT |
| qPCR_*mph(A)_*P2 | GCGAGGTACTCTTCGTTACCC |
| qPCR_ *mphR(A)_*P1 | AGCGGAGTAGCAAAGGAGGT |
| qPCR_ *mphR(A)_*P2 | CTGGAGCTCGTACCAGGAGA |
| qPCR_ *mrx_*P1 | GTAGATGCAGGACGCCAAGT |
| qPCR_ *mrx_*P2 | CTGGCAACTGTGAAAGCAGA |
| qPCR_ *chrA_*P1 | GTAATGGCAGCACAACGTGA |
| qPCR_ *chrA_*P2 | ATTGTTCAAGCCAGCGAAAG |
| qPCR_ *sul1_*P1 | GTCTAAGAGCGGCGCAATAC |
| qPCR_ *sul1_*P2 | CTCACCGAGGACTCCTTCTTC |
| qPCR_ *aadA2_*P1 | ACCAAGGCAACGCTATGTTC |
| qPCR_ *aadA2_*P2 | TCCGCGCTATAGAAGTCACC |
| qPCR_ *dhfr7_*P1 | AGCTTGAATGGTTTCGGTTG |
| qPCR_ *dhfr7_*P2 | CGAACTCGGCAATGAACTCT |
| qPCR_ *int_*P1 | CGTAGAACAAGCAGGCATCA |
| qPCR_ *int_*P2 | TGCGTGTAAATCATCGTCGT |
| qPCR_ *tem-1_*P1 | CCAGAAACGCTGGTGAAAGT |
| qPCR_ *tem-1_*P2 | TACCGCACCACATAGCAGAA |
| qPCR_ *ctx-m-15_*P1 | CAGCTGGTGACATGGATGAA |
| qPCR_ *ctx-m-15_*P2 | CTTAGGTTGAGGCTGGGTGA |
| qPCR_ *aac(6’)-Ib-cr _*P1 | GGAGTACGCGGAATAGACCA |
| qPCR_ *aac(6’)-Ib-cr _*P2 | GGTTACGGTACCTTGCCTCTC |
| qPCR_ *oxa-1 _*P1 | GCAAATGGCACCAGATTCA |
| qPCR_ *oxa-1 _*P2 | AATTGCATCCACGTCTTTGG |
| qPCR_ *tetA_*P1 | CCTCTTCACGGCGATCTATG |
| qPCR_ *tetA_*P2 | GGCAGGCAGAGCAAGTAGAG |
| qPCR_ *tetR_*P1 | CCGAATGCGTATGATTCTCC |
| qPCR_ *tetR_*P2 | GTCTGACGACACGCAAACTG |
|  |  |

^#^ Del_IS26_P1 together with Del_IS26_P2 will enable the substitution of IS26 elements that are tandem oriented (🡪 🡪). P1 and P3 will substitute IS26 elements oriented toward each other (🡪 🡨). P7 and P8 will substitute IS*26* elements oriented away from each other (🡨 🡪).

Letters in **bold** denote signal sequence.

Letters underlined denote the beta-lactamase coding region.
