# Supplementary material for: The Role of Antibiotic Resistance Genes in the Fitness Cost of Multiresistance Plasmids
Source: mBio. 2022 Jan 18;13(1):e03552-21. doi: 10.1128/mbio.03552-21 (PMC8764527; doi:10.1128/mbio.03552-21)
Supplement: TEXT S1 [file mbio.03552-21-s0001.docx]

**Supplementary Materials and methods**

*Protein extraction for proteomic analysis*

Cell pellets were homogenized using a FastPrep®-24 instrument (Matrix B (blue); MP Biomedicals, OH, USA) for 4 repeated 40 seconds cycles at 6.5 m/s in 120 µl of the buffer containing 2% sodium dodecyl sulfate and 50mM triethylammonium bicarbonate (TEAB). Samples were centrifuged at 13 000 rpm for 10 min and the supernatants were transferred to clean tubes. The lysis tubes were washed with 80 µl of the lysis buffer, centrifuged at 13 000 rpm for 10 min, the supernatants were combined with the corresponding lysates from the previous step. Protein concentration in the combined lysates was determined using Pierce™ BCA Protein Assay Kit (Thermo Scientific) and the Benchmark™ Plus microplate reader (BIO-RAD) with bovine serum albumin (BSA) solutions as standards.

*Tryptic digestion and Tandem Mass Tag (TMT) labelling*

Aliquots containing 30 µg of total protein were taken from each sample and reduced at 56°C for 30 min in the lysis buffer with DL-dithiothreitol (DTT) at 100 mM final concentration and incubated. The reduced samples were processed using the modified filter-aided sample preparation (FASP) method^35^. In short, reduced samples were diluted to 500 µl by addition of 8M urea, transferred onto Nanosep 10k Omega filters (Pall Life Sciences) and washed 2 times with 200 µl of 8M urea. Alkylation of the cysteine residues was performed using 10 mM methyl methanethiosulfonate (MMTS) diluted in digestion buffer (1% sodium deoxycholate (SDC), 50 mM TEAB) for 30 min at room temperature and the filters were then repeatedly washed with digestion buffer. Trypsin (Pierce Trypsin Protease, MS Grade, Thermo Fisher Scientific) in digestion buffer was added in a ratio of 1:100 relative to total protein mass and the samples were incubated at 37°C for 3h; another portion of trypsin (1:100) was added and incubated overnight. The peptides were collected by centrifugation and labelled using Tandem Mass Tag (TMT) reagents (Thermo Scientific) according to the manufacturer’s instructions. The labelled samples were combined, pooled samples were concentrated using vacuum centrifugation and SDC was removed by acidification with 10% TFA and centrifugation.

The combined TMT-labeled sample was fractionated into 40 primary fractions by basic reversed-phase chromatography (bRP-LC) using a Dionex Ultimate 3000 UPLC system (Thermo Fischer Scientific). Peptide separations were performed using a reversed-phase XBridge BEH C18 column (3.5 μm, 3.0x150 mm, Waters Corporation) and a linear gradient from 3% to 40% solvent B over 17 min followed by an increase to 100% B over 5 min. Solvent A was 10 mM ammonium formate buffer at pH 10.00 and solvent B was 90% acetonitrile, 10% 10 mM ammonium formate at pH 10.00. The primary fractions were concatenated into final 20 fractions (1+21, 2+22, … 20+40), evaporated and reconstituted in 15μl of 3% acetonitrile, 0.2% formic acid for nLC MS analysis.

*LC-MS/MS Analysis*

The fractions were analyzed on an Orbitrap Fusion Tribrid mass spectrometer interfaced with Easy-nLC1200 liquid chromatography system (both Thermo Fisher Scientific). Peptides were trapped on an Acclaim Pepmap 100 C18 trap column (100 μm x 2 cm, particle size 5 μm, Thermo Fischer Scientific) and separated on an in-house packed analytical column (75 μm x 30 cm, particle size 3 μm, Reprosil-Pur C18, Dr. Maisch) using a linear gradient from 5% to 32% B over 75 min followed by an increase to 100% B for 5 min, and 100% B for 10 min at a flow of 300 nL/min. Solvent A was 0.2% formic acid in water and solvent B was 80% acetonitrile, 0.2% formic acid. MS scans was performed at 120 000 resolution, m/z range 380-1380, MS/MS analysis was performed in a data-dependent, with top speed cycle of 3 s for the most intense doubly or multiply charged precursor ions. Most intense precursors were fragmented in MS2 by collision induced dissociation (CID) at 35 collision energy with a maximum injection time of 50 ms, and detected in the ion trap followed by multinotch (simultaneous) isolation of the top 7 MS2 fragment ions, with m/z 400-1400, selected for fragmentation (MS3) by higher-energy collision dissociation (HCD) at 65% and detection in the Orbitrap at 50 000 resolution, m/z range 100-500. Precursors were isolated in the quadrupole with a 0.7 m/z isolation window and dynamic exclusion within 10 ppm during 45 seconds was used for m/z-values already selected for fragmentation. After the database search on the LC-MS files, the m/z and z for the identified peptides was exported and used to create a targeted exclusion list. Each fraction was then re-analyzed using the method described above, with the added targeted exclusion.

*Proteomic Data Analysis*

Identification and relative quantification were performed using Proteome Discoverer version 2.2 (Thermo Fisher Scientific). The reference *E. coli* K12 database was downloaded from Uniprot (October 2017) and supplemented with the plasmid sequences and common proteomic contaminants (4599 sequences in total). The database search was performed using the Mascot search engine v. 2.5.1 (Matrix Science, London, UK) with MS peptide tolerance of 5 ppm and fragment ion tolerance of 0.6 Da. Tryptic peptides were accepted with 1 missed cleavage; methionine oxidation was set as a variable modification, cysteine methylthiolation, TMT-6 on lysine and peptide N-termini were set as fixed modifications. Percolator was used for PSM validation with the strict FDR threshold of 1%. Quantification was performed in Proteome Discoverer 2.2. TMT reporter ions were identified in the MS3 HCD spectra with 3 mmu mass tolerance, and the TMT reporter intensity values for each sample were normalized within Proteome Discoverer 2.2 on the total peptide amount. Only the unique identified peptides were taken into account for the relative quantification.
